# Impact of Lyophilized Milk Kefir-Based Self-Nanoemulsifying System on Cognitive Enhancement via the Microbiota–Gut–Brain Axis

**DOI:** 10.3390/antiox13101205

**Published:** 2024-10-07

**Authors:** Mai M. Anwar, Amira A. Boseila, Abeer A. Mabrouk, Abdelfattah A. Abdelkhalek, Amr Amin

**Affiliations:** 1 Department of Biochemistry, National Organization for Drug Control and Research (NODCAR)/Egyptian Drug Authority (EDA), Giza 12654, Egypt; mainwr@hotmail.com (M.M.A.);; 2 Department of Pharmaceutics, National Organization for Drug Control and Research (NODCAR)/Egyptian Drug Authority (EDA), Giza 12654, Egypt; amira.hamada@su.edu.eg; 3 Department of Pharmaceutics and Industrial Pharmacy, Faculty of Pharmacy, Sinai University, Kantara Branch, Ismailia 41636, Egypt; 4 Department of Basic Science, Faculty of Physical Therapy, Al Hayah University, Cairo 11835, Egypt; 5 College of Medicine, University of Sharjah, Sharjah 27272, United Arab Emirates

**Keywords:** inflammatory bowel disorders, Caraway oil, licorice extract, cognitive decline, self-nanoemulsifying self-nanosuspension, gut–brain axis disorder, milk kefir

## Abstract

Chronic inflammatory bowel disorders (IBDs) are characterized by altered intestinal permeability, prompting inflammatory, oxidative stress, and immunological factors. Gut microbiota disorders impact brain function via the bidirectional gut–brain axis, influencing behavior through inflammatory cascades, oxidative stress, and neurotransmitter levels. This study highlights the potential effect of integrating lyophilized milk kefir alone and lyophilized milk kefir as solid carriers loaded with a self-nanoemulsifying self-nanosuspension (SNESNS) of licorice extract on an induced chronic IBD-like model in rats. Licorice-SNESNS was prepared by the homogenization of 30 mg of licorice extract in 1 g of the selected SNEDDS (30% Caraway oil, 60% Tween 20, and 10% propylene glycol (*w*/*w*)). Licorice-SNESNS was mixed with milk kefir and then freeze-dried. Dynamic TEM images and the bimodal particle size curve confirmed the formation of the biphasic nanosystems after dilution (nanoemulsion and nanosuspension). Daily oral administration of lyophilized milk kefir (100 mg/kg) loaded with SNESNS (10 mg/kg Caraway oil and 1 mg/kg licorice) restored normal body weight and intestinal mucosa while significantly reducing submucosal inflammatory cell infiltration in induced rats. Importantly, this treatment demonstrated superior efficacy compared to lyophilized milk kefir alone by leading to a more significant alleviation of neurotransmitter levels and improved memory functions, thereby addressing gut–brain axis disorders. Additionally, it normalized fecal microbiome constituents, inflammatory cytokine levels, and oxidative stress in examined tissues and serum. Moreover, daily administration of kefir-loaded SNESNS normalized the disease activity index, alleviated histopathological changes induced by IBD induction, and partially restored the normal gut microbiota. These alterations are associated with improved cognitive functions, attributed to the maintenance of normal neurotransmitter levels and the alleviation of triggered inflammatory factors and oxidative stress levels.

## 1. Introduction

The gut microbiome, often referred to as the “forgotten organ”, has gained recognition for its profound influence on host physiology. Its composition varies based on the state of the individual and the severity of the disease. Recent estimates suggest that the gastrointestinal tract houses more than 10^15^ microorganisms, with the number of bacterial cells surpassing the number of host cells by a factor of ten. Moreover, the genetic materials of the microbiome outnumber the host genome by a factor of 100 or more [1]. The gut microbiota plays a vital role in regulating the gut–brain axis by secreting or triggering metabolites and proteins that facilitate the release of gut hormones and neuropeptides and the synthesis of neurotransmitters and their precursors. The human gut harbors thousands of bacteria, and their presence is crucial for bidirectional communication between the gut and the brain. These bacteria regulate immunological, endocrine, and neurological pathways, emphasizing their importance in gut–brain communication. Compared to individuals without similar diseases, patients with cognitive and neurodegenerative disorders display unique gut microbiomes and gut–brain axis anomalies. Additionally, the gut microbiome influences signals that shape immune functions, impacting normal metabolism and human physiology [2,3]. Inflammatory bowel disease (IBD) is an immune system-mediated inflammatory disorder primarily affecting the gastrointestinal tract. Conditions such as Crohn’s disease (CD) and ulcerative colitis (UC) are encompassed within IBD. These disorders are linked to various contributing factors, including genetic, environmental, inflammatory, microbial, and oxidative stress conditions [4]. The intestinal mucosal barrier (IMB) serves as the primary mechanical, microbial, and immune barrier, preventing the entry of pathogenic molecules into the bloodstream. Individuals diagnosed with UC consistently exhibit intestinal barrier dysfunction. This implies that UC is invariably associated with substantial damage to the integrity of the IMB, often due to an abnormal increase in certain types of pathogenic bacteria or a deficiency in beneficial bacteria [5]. Even minor damage to the intestinal barrier leads to a significant increase in intestinal permeability.

Recent research has underscored the significant impact of IBD on cognitive functions, revealing that patients with IBD are at an increased risk of experiencing cognitive decline and memory impairment. This association is primarily attributed to heightened oxidative stress, intestinal injury, and disruptions in the gut–brain axis [6]. The gut microbiota, which plays a crucial role in maintaining intestinal homeostasis, becomes dysbiotic in IBD, triggering local inflammatory responses and compromising the integrity of the intestinal barrier. This dysbiosis leads to systemic inflammation, which can cross the blood–brain barrier (BBB) and induce neuroinflammation [7]. Disruption of the intestinal mucosal immune bidirectional system, often associated with UC, leads to an overproduction of proinflammatory cytokines and oxidative stress [8]. These inflammatory cytokines can reach the brain through systemic circulation, resulting in behavioral changes, cognitive decline, apoptosis, and neuroinflammation. Increased levels of neurotoxic substances such as nitric oxide (NO) and reactive oxygen species (ROS) contribute to this neuroinflammatory response [8,9,10,11]. Thus, the gut–brain axis describes a unique bidirectional communication system between the central nervous system (CNS) and the gastrointestinal tract. This complex axis involves various systems, including the autonomic nervous system, neuroimmune system, CNS, neuroendocrine system, and gut microbiota. Any modulation in the gut microbiota is suggested to be directly correlated with abnormal brain function and mental disorders [6,12]. The primary bidirectional link in the gut–brain axis involves the vagus nerve, often referred to as the “crossed neuroimmune interactions nerve” [13]. Consequently, dysbiosis in the gut microbiome is suggested to directly contribute to the progression of behavioral changes, cognitive decline, and neurodegenerative diseases [8,14].

Probiotics, living microorganisms found in fermented products, offer several health benefits when consumed regularly [15,16]. Both Lactobacillus and Bifidobacterium, two significant lactic acid bacteria species, have been utilized as probiotics since Elie Metchnikoff’s research in 1907 to treat a range of illnesses. These bacteria produce active metabolites such as vitamins, organic acids, and other bioactive compounds [17]. Probiotics support intestinal microbiota homeostasis by improving mucin synthesis, controlling cytokines, and acting as immunomodulators and potent anti-inflammatory agents [15,17]. Given that the etiology of IBDs involves various immunological and inflammatory factors affecting the diversity and composition of the intestinal microbiota, alternative natural therapies in novel formulations are needed to effectively combat IBDs [2,18]. Whereas, nanoformulations significantly enhance the overall bioavailability and stability of given bioactive compounds, thereby enhancing their anti-inflammatory, antioxidant, and immunomodulatory properties [19]. By increasing the solubility and absorption of administrated agents, the nanoemulsified system facilitates more efficient delivery to the intestinal mucosa, which plays a critical role in modulating the gut microbiota, reducing intestinal inflammation, and restoring mucosal integrity [19]. This advanced delivery system not only targets local inflammation in the gut but also mitigates systemic effects, including those related to the gut–brain axis, thereby offering a comprehensive therapeutic approach to IBD management. On the other hand, current treatments often involve the use of corticosteroids, immunosuppressants, and biologics, which can have significant side effects such as increased susceptibility to infections, liver toxicity, and bone marrow suppression. Additionally, these medications may not be effective for all patients and can lose efficacy over time, necessitating long-term, high-dose treatments that further increase the risk of adverse effects [20]. Thus, these challenges and previous findings have led to the exploration of probiotics and adjuvant medicines in novel pharmaceutical forms designed to enhance their administration, absorption, and bioavailability. Kefir is a sort of milk product that has undergone fermentation and is made up of various fungi, yeast, and bacteria that create grain structures. It originated in the Russian Caucasus mountains centuries ago and has various health benefits [21]. In addition to improving the gut microbiota composition, kefir-fermented milk has antioxidant, anti-inflammatory, antimycotic, antitumor, and immunomodulatory effects [22,23,24]. The immune-modulatory action of kefir grains may be related to its direct action on the microbiota and/or its indirect effect through the bioactive compounds and metabolites produced during the fermentation of starter grains [21]. These metabolites are suggested to have direct healing effects on IBDs and gut–brain axis disorders due to their wide range of active biological properties [25].

Self-nanoemulsifying drug-delivery systems (SNEDDSs) are characterized by high stability and promising bioavailability of drug molecules. The main advantage of these systems is their ability to generate nanodroplets that have great potential in pharmaceutical applications with easy industrial scale-up due to simple formulation processes [26,27]. Self-nanoemulsifying self-nanosuspension (SNESNS) is a dual solubility enhancement system that combines the advantages of both nanoemulsions and nanosuspensions. SNESNS addresses the drawbacks of self-nanoemulsifying drug-delivery systems, such as the inability to load high drug doses. SNESNS is composed of an isotropic mixture of oil, a surfactant, and a cosurfactant. The drug is loaded in this system in two forms: dissolved and suspended nanoparticles, which spontaneously form nanoemulsions and nanosuspensions once they are diluted with aqueous fluids [28]. However, because of their oily liquid characteristics, these systems have several disadvantages when they are liquid, such as limited thermodynamic stability and low mobility [29]. To overcome the disadvantages related to liquid SNEDDSs, several techniques have been followed to solidify them. These include their adsorption on solid porous carriers using conventional adsorption techniques, spray drying, lyophilization, and pelletization [30]. For solidification, the most commonly used solid carriers are silicon dioxide (SiO_2_), lactose, microcrystalline cellulose, magnesium stearate, and talc [31]. Considering the multiple health benefits of probiotics obtained from lyophilized milk kefir, an attempt has been made to explore lyophilized milk kefir as a solid carrier for the conversion of liquid SNESNS to solid SNESNS.

Egyptian Caraway oil is composed mainly of limonene, β-selinene, β-elemene belemene, and caryophyllene oxide. These bioactive compounds exhibit a number of advantageous biological characteristics, including antibacterial, antioxidant, anti-inflammatory, and anticonvulsant activities [32]. In our study, Caraway oil and licorice extract were not used as therapeutic agents. They were otherwise used as adjuvant agents to be administered in combination with a lyophilized probiotics food supplement that proved its homeostasis effect on the gut microenvironment. For those not accustomed to consuming kefir or other fermented dairy products, introducing kefir probiotics and yeasts may initially cause gastrointestinal discomfort, such as bloating, gas, or stomach ache, as the gut microbiome adjusts to the new microbial population [33]. According to the literature, Caraway (*Carum carvi*) oil is composed of various bioactive compounds, with the primary ones being Carvone (50–70%), Limonene (20–30%), Pinene (5–10%), Myrcene (3–5%), and Linalool (1–3%) [34,35]. Caraway oil possesses carminative gas-relieving effects and antispasmodic properties, which can help alleviate symptoms of bloating, abdominal discomfort, and functional gastrointestinal disorders. The combination of Caraway oil with milk kefir aims to counteract the potential gastrointestinal distress caused by probiotics [36,37]. Licorice (Glycyrrhiza glabra) has been widely utilized for many years in folk medicine. Licorice and its derived compounds show antiallergic, antibacterial, antiviral, anti-inflammatory, and antitumor effects. These pharmacological properties are exploited for the management of inflammatory disorders [38]. The primary bioactive compounds found in licorice (Glycyrrhiza) extract are Glycyrrhizin (4–20%), flavonoids (such as liquiritin, isoliquiritin, liquiritigenin, and isoliquiritigenin), triterpenes (including glycyrrhetinic acid, 18β-glycyrrhetinic acid, and oleanolic acid), phenolic compounds (like ferulic acid, p-coumaric acid, and caffeic acid), and chalcones (such as echinatin and licochalcone A) [39,40]. To address the limitations of administering probiotics and SNESNS in liquid dosage forms, lyophilized milk kefir grain (L-MKG) was prepared and used as a solid carrier to be loaded with SNESNS of Caraway oil and licorice extract (L-MKG/SNESNS). This system has the potential to be a solid self-nanoemulsifying system with increases in solubility, absorption, effectiveness, and stability for the effective oral delivery of hydrophobic biologically active moieties [41,42].

The primary objective of the current study is to assess the inflammatory processes, triggered cytokines, and oxidative stress production in an IBD-like animal model. In order to highlight the ensuing gut–brain axis problem, this study focuses on behavioral abnormalities, cognitive decline, and decreased neurotransmitter levels in conjunction with different forms of histopathological damage to the brain. This investigation also evaluates the potential impact of the administration of L-MKG and L-MKG/SNESNS on the alteration of the gut microbiota, with a concurrent assessment of its contribution to mitigate inflammation in the intestinal tract. Furthermore, here we will shed more light on the therapeutic effects of L-MKG/SNESNS, proposing that its administration exerts anti-inflammatory, antioxidant, and immunomodulatory effects in a rat model of IBD-like conditions. The anticipated outcome is the restoration of gut–brain axis disorders and the induction of healing within the intestinal tract.

In conclusion, the goal of this study is to gain a thorough understanding of the complex interactions that exist between gut–brain axis disorders, chronic IBD-like conditions, and the potential therapeutic benefits of L-MKG/SNESNS in treating behavioral manifestations, oxidative stress, and inflammation in the context of intestinal disorders.

## 2. Materials and Methods

### 2.1. Kefir Grain and Chemicals

A milk kefir grain starter was obtained from a culture of health (150 Morrisville, NC, 27560-8591 USA). Dextran sulfate sodium (DSS) and all other required chemicals of high analytical grade were purchased from Merck (Darmstadt, Germany) and Sigma–Aldrich (St. Louis, MO, USA). Caraway oil was purchased from Nefertari Oil (Cairo, Egypt); Tween 20 and propylene glycol (PG) were purchased from Sigma Aldrich (St. Louis, MO, USA); and licorice extract was a gift from the Egyptian Pharmaceutical Company (Alexandria, Egypt).

### 2.2. Lyophilized Milk Kefir Grains (L-MKGs)

Lyophilized milk kefir grains (L-MKGs) were prepared through a multistep process. Initially, 20 g of a milk kefir grain starter was added to fully pasteurized milk and allowed to ferment at 25 °C for 24 h. After fermentation, the milk was filtered through a sterile plastic sieve to separate it from the grains. To ensure the grains were clean for the next batch, they were thoroughly washed with pasteurized milk before each new inoculation [22,23,24]. The starter of the processed milk kefir grains was then stored under vacuum conditions at 1 Pa (Büchi Labortechnik AG, Flawil, Switzerland), maintaining a temperature of −39 °C. This storage method preserved the grains’ quality until they were needed. Finally, the grains were lyophilized using a freeze-dryer (Alpha 2–4, CHRIST, Osterode am Harz, Germany), resulting in lyophilized milk kefir grains (L-MKGs). This freeze-drying process created a stable, dry form of the grain that retains its properties for long-term use in experiments or other applications.

### 2.3. Construction of Ternary Phase Diagrams

The aim of ternary phase diagram construction was to optimize the ratios of oil, surfactant, and co-surfactant in the formulation of the self-nanoemulsifying system (SNES) [43,44,45]. The diagrams help identify the regions where stable and effective emulsions can be formed, ensuring the solubilization and bioavailability of the active compounds. This step is crucial for the development of a robust and reproducible formulation with enhanced therapeutic efficacy. The ternary phase diagram was created using Caraway oil (obtained from Agricultural Research Center, Giza, Egypt), Tween 20 (as a surfactant), and propylene glycol (as a cosolvent) purchased from Sigma–Aldrich (St. Louis, MO, USA) to detect the areas where each observed point could directly form a nanoemulsion upon dilution. Required mixtures were prepared with varying concentrations of these components, followed by the construction of the ternary phase diagram. Each point on the diagram underwent testing for its ability to self-emulsify by diluting 1 g of the mentioned corresponding ternary mixture with distilled water up to 200 mL in a glass beaker. The mixture was then stirred magnetically at 37 °C for 5 min. Visual observation and inspection were employed to detect any phase separation in the diluted mixtures. Dispersion with a transparent appearance was categorized as falling within the nanoemulsion region [46].

### 2.4. Preparation of SNESNS from Licorice Extract

A self-nanoemulsifying formulation was selected from the ternary phase diagram to be used to prepare licorice-SNESNS. The formula composed of 30% Caraway oil, 60% Tween 20, and 10% propylene glycol (*w*/*w*) was prepared as follows: for each 150 gm of the formulation, 45 g of Caraway oil, 90 g of Tween 20, and 15 g of propylene glycol were mixed, and then 100 mL of the prepared mixture was mixed with 3 g of licorice extract (to yield a concentration of 3 mg/mL) obtained from (Sekem Company, Cairo, Egypt) with the aid of homogenization (CAT X520, Ballrechten-Dottingen, Germany) for 5 min. The formula was prepared and subjected to further characterization [47].

### 2.5. Preparation of Solid SNESNS from Licorice Extract (L-MKG/SNESNS)

Ten milliliters of the prepared licorice -SNESNS formula was added dropwise to 25 mL of the milk kefir dispersion on a magnetic stirrer until a homogenous dispersion was obtained. The whole mixture was then frozen at −20 °C and lyophilized under reduced pressure to obtain lyophilized milk kefir-loaded licorice-SNESNS in powder form.

### 2.6. Physico-Chemical Characterization of Licorice-SNESNS and L-MKG/SNESNS

#### 2.6.1. Particle Size, Polydispersity Index (PDI), and Zeta Potential of L-MKG/SNESNS and Licorice-SNESNS

The mean particle size and PDI of the prepared licorice-SNESNS and solid L-MKG/SNESNS were measured using a Malvern Zetasizer 3000 (Grovewood Road, Malvern, Worcestershire, WR14 1XZ, UK) with a dynamic light-scattering mechanism. The measurements were performed in a quartz cuvette after each sample was diluted 100-fold with deionized water.

#### 2.6.2. TEM

The morphologies of the prepared solid L-MKG/SNESNS and licorice-SNESNS were observed by TEM (JEOL, JEM-1230, Tokyo, Japan) after dilution with deionized water. A drop of the diluted samples was placed on a copper grid and stained with a 2% phosphotungstic acid solution Merck (Merck KGaA, Darmstadt, Germany). The excess staining agent was removed with the aid of filter paper, and then the grid was left to completely dry at ambient temperature.

### 2.7. Animals

Thirty healthy, pathogen-free male albino rats, each with a body weight ranging from 180 to 200 g, were obtained from the animal house of the National Organization of Drug Control and Research (NODCAR). The rats had unrestricted access to standard laboratory chow and water during the whole experimental design and were subjected to specific environmental conditions, including a temperature of 25 ± 1 °C, humidity maintained between 50 and 70%, and a 12 h light–dark cycle. The study was approved and adhered to guidelines for the animal ethical committee of the National Organization of Drug Control and Research (NODCAR) under the reference number (NODCAR/II/6/2023) and followed the principles of the 3Rs (Refine–Reduce–Replace). Before the experiment commenced, the rats were allowed to acclimatize for one week to adapt to their surroundings. Throughout the study, all animals were provided unrestricted access to food and water. The ethical considerations and principles employed aimed to ensure the well-being and humane treatment of the experimental animals.

### 2.8. Experimental Design

The thirty male albino rats were equally and randomly divided into the following groups each with 6 rats (n = 6):

1. Group 1 (HCG): This group served as the control for the Healthy Control Group (HCG) and consisted of rats administered water instead of the designed formulated milk kefir grains.
2. Group 2 (A-IBD-like model): This group included rats administered dextran sulfate sodium (DSS) at a concentration of 5% daily by oral gavage for 10 days to establish an acute IBD-like model [48,49,50,51,52,53,54]. The aim of conducting this group was to serve as an intermediate phase in our study. Its primary purpose is to demonstrate that, after ten days, the disease progresses to a chronic state in a cascading process. It was crucial to conduct the A-IBD-like model group to confirm the establishment and progression of the chronic IBD model before we evaluate the therapeutic effects of L-MKG and L-MKG/SNESNS treatments. By including this group, we can ensure that the chronic IBD model is accurately established, providing a solid foundation for assessing the efficacy of our treatments in the chronic phase of the disease. This intermediate phase is essential for understanding the cascade of disease progression and validating the chronic state as the target for our therapeutic interventions.
3. Group 3 (C-IBD-like model): This group included rats administered DSS (5%) daily by oral gavage for 16 days to establish a chronic IBD-like model [48,49,50,51,52,53,54,55,56].
4. Group 4 (C-IBD-like model+ L-MKG): This group included induced chronic IBD-like model rats administered DSS (5%) daily by oral gavage for 16 days [48,49,50,51,52,53,54,55,56], followed by treatment with lyophilized milk kefir grains (L-MKGs) for 10 days at a dose of 150 mg/kg via oral gavage [57]. The exact bioactive constituents in milk kefir grains have been previously demonstrated in other studies [58,59,60].
5. Group 5 (C-IBD-like model+ L-MKG/SNESNS): This group included induced chronic IBD-like model rats administered DSS (5%) daily by oral gavage for 16 days [48,49,50,51,52,53,54,55,56], followed by treatment with lyophilized milk kefir grains (L-MKGs) loaded with SNESNS of licorice extract daily for 10 days at a dose of 100 mg/kg via oral gavage [57]. Each 100 mg of L-MKG/SNESNS contains 10 mg of Caraway oil and 1 mg of licorice extract according to our prepared formulation.

### 2.9. Body Weight

The body weight of each rat in all five groups was documented at the conclusion of the experiment and compared with their initial weights recorded before the commencement of the experimental design. This approach was chosen to effectively assess the overall impact of the treatments on the rats’ well-being. Monitoring body weight at the start and end of the experiment provides a clear and direct measurement of weight change, which is a critical indicator of the rats’ health and response to the treatments. By comparing the initial and final weights, we can accurately evaluate the efficacy and any potential side effects of the treatments. This method is efficient and minimizes the handling of animals, reducing stress that could confound the experimental results. Additionally, this approach aligns with ethical considerations for animal welfare by limiting the frequency of interventions. Thus, the changes in body weight were documented after the experiment for each rat in all five groups, and the values were compared with their initial weights before the commencement of the experimental design [61,62].

### 2.10. Disease Activity Index

The disease activity index (DAI) was utilized to evaluate the severity of colitis in the rat model. The DAI is a composite score based on three clinical parameters: weight loss, bleeding severity, and stool consistency. Weight loss was assessed and scored as follows: 0 points for no weight loss, 1 point for 5–10% weight loss, 2 points for 11–15% weight loss, 3 points for 16–20% weight loss, and 4 points for more than 20% weight loss. Bleeding severity was recorded as 0 points for no bleeding and 4 points for the presence of bleeding. Stool consistency was classified and scored as 0 points for normal stool, 2 points for loose stool, and 4 points for diarrhea. The DAI is calculated by summing the scores from these three categories, with the total score reflecting the overall disease severity. Higher DAI scores indicate more severe colitis. The specific methodology and scoring system used for this assessment are consistent with established practices described in the literature [63,64,65,66,67,68].

### 2.11. Sample Collection, Processing, and Staining

One day following the conclusion of the experimental design and one day prior to sacrifice through cervical dislocation, blood samples were collected from the retroorbital venous plexus and subsequently centrifuged at 1000× *g* for 10–15 min at 4 °C. The resulting serum was then stored at −80 °C for future use. Stool samples were obtained from each rat in the respective groups on the same day before sacrifice. The stools were weighed and inoculated in 1% saline for subsequent biochemical detection. The brain, intestine, stomach, and colon were isolated and harvested from rats under the effect of isoflurane anesthesia (2–3% in 100% oxygen) (Acdima international, Cairo, Egypt) [69]. The brain, including the cortex and hippocampus, as well as the intestine, stomach, and colon, were then harvested. The colon’s length was recorded. The isolated tissues were processed as follows: the brain (including the cortex and hippocampus) was fixed in 4% paraformaldehyde (PFA) (Sigma-Aldrich, St. Louis, MO, USA) and embedded in paraffin wax for hematoxylin-eosin (H&E) staining (Abcam Inc. 152 Grove Street, Waltham, MA, USA) and histopathological analysis. The small intestine and stomach were also fixed in 4% PFA (Sigma-Aldrich, St. Louis, MO, USA), embedded in paraffin, and subjected to H&E staining. Additionally, sections of the small and large intestines were processed using trichrome staining (Abcam Inc., Waltham, MA, USA) to evaluate the amount or density of collagen fibers. This assessment helps indicate pathological changes or conditions affecting the tissue compared to the control group, following the methodologies described by Araruna et al. and Matei-Lațiu et al. [70,71]. Tissue samples were divided into two portions: one for preparation of tissue homogenates for biochemical analysis and the other for histopathological examination.

### 2.12. Determination of Luminal Bacterial Concentrations

Cecal contents were collected from euthanized rats, and 1 mL of these contents was immediately weighed and prepared for analysis. The cecal samples were serially diluted in pre-reduced thioglycolate broth (Sigma–Aldrich, St. Louis, MO, USA) to maintain anaerobic conditions. From each dilution, 100 µL was plated on pre-reduced, anaerobically sterilized agar plates, which were incubated in an anaerobic chamber with an atmosphere of 5% CO_2_, 10% H_2_, and 85% N_2_. Additionally, blood agar plates were used to culture aerobic bacteria. The agar plates were incubated at 37 °C, with aerobic cultures assessed after 2 days and anaerobic cultures after 6 days. Colonies were counted to determine bacterial concentrations in the cecal contents [72,73]. Detailed information regarding the primary isolation media and colonial appearance is available in the Supplementary File S1.

### 2.13. Identification of Colitis Bacteria

To identify colitis-associated bacteria, the cecal contents were analyzed using the same plated samples and incubation conditions described in Section 2.12. After incubation, colonies were examined and characterized based on their morphology and growth patterns. Specific bacterial identification was performed using standard biochemical tests and, where applicable, using phase-contrast microscopy and a Neubauer counting chamber to accurately determine the presence of bacteria associated with colitis. This comprehensive approach allowed for detailed analysis of both aerobic and anaerobic bacterial populations in the cecal contents [72,73].

#### Methods

The identification of colitis-associated bacteria was carried out using a systematic approach. Initially, a quick oxidase test was performed on the cultured bacteria to determine the presence of cytochrome c oxidase. A single isolated colony from a pure culture was then selected and suspended in sterile distilled water. The API20E Biochemical Test Strip (BioMérieux, Marcy-l’Etoile, France) was used for further identification, which includes dehydrated bacterial media and biochemical reagents in 20 separate compartments. Each compartment was filled with the bacterial suspension using a Pasteur pipette, and sterile oil was added to specific compartments as required. The test strip was incubated at 37 °C for 18 to 24 h. For result interpretation, color changes in the compartments were observed after incubation. Some compartments required additional reagents for color development: ferric chloride for TDA, Kovacs reagent for IND, and a combination of 40% KOH (VP reagent 1) and α-naphthol (VP reagent 2) for VP. The results were recorded using the API Reading Scale, where positive or negative marks were made for each test. The scores from triplets of wells were summed to generate a 7-digit code, which was used to identify the bacterial species by referencing the API catalog [74,75].

### 2.14. Behavioral Studies

In the behavioral studies, two distinct tests were employed to assess various aspects of rats’ behavior:

The Tail Suspension Test (TST) is a well-established method primarily used to measure the duration of immobility in rodents when they are suspended by their tails. This immobility is considered indicative of behavioral despair, a model often used to evaluate antidepressant-like effects and depressive behaviors. While the TST is traditionally associated with assessing depressive behaviors, there is emerging evidence that it can also provide insights into broader cognitive aspects. Specifically, the TST can reveal how neuroimmune responses and emotional and motivational states influence cognitive processing. For instance, stress-related alterations in the neuroimmune system can affect both mood and cognitive functions, including memory and decision-making. This interplay suggests that the TST may reflect not only behavioral despair but also underlying changes in cognitive processes influenced by emotional and neuroimmune factors [76,77,78,79,80]. TST is traditionally more common in smaller rodents such as mice, and the recent literature supports its application in larger rodents, including rats [81,82,83,84,85]. After completing the experimental phase, the TST was conducted on all rats the day before blood collection and tissue isolation. This involved placing each rat in a quiet, isolated square box equipped with a hook, suspending approximately 1 cm of its tail about 50 cm above the ground. The total suspension period was approximately 6 min, with the first 60 s designated as the adaptive latency time. The immobility period, recorded as the time when the rat ceased struggling, served as an indicator of anxiety and depression-like behavior. A shorter immobility period suggested greater susceptibility to these behavioral traits [81,82,83,84,85].

In the Y-maze test, an enclosed maze test was utilized to evaluate spatial memory and study cognitive functions, including those associated with the hippocampal region and cognitive decline. This test capitalizes on the inherent exploratory tendencies of animals, which naturally seek out new environments, making it ideal for assessing cognitive capabilities. It is particularly effective at identifying impairments in spatial recognition memory, which can be caused by factors like stress, neurological disorders, or pharmacological interventions [86,87,88]. The maze consisted of three arms forming a Y-shape, with two arms designated as goal arms containing rewards. Doors at the entrance of each arm either confined the rat to specific arms or prevented entry into certain arms. The Y-maze leverages the rat’s motivation to explore the surroundings and seek rewards. Healthy rats demonstrated the ability to remember previously entered arms, alternating to the opposite arm in subsequent trials. In contrast, aged and stressed rats exhibited difficulty remembering the correct choice of arms. Prior to the main test, a pretraining phase was conducted three times to familiarize the rats with the maze. The spontaneous alternation percentage (SAP) was then calculated from the total number of rat alternations divided by the number of arm entries minus 2, multiplied by 100: % SAP = [(number of rat alternations)/(number of arm entries − 2)] × 100. After each trial in both tests, the testing apparatus was consistently cleaned with 10% ethanol to maintain a standardized testing environment [81].

### 2.15. Myeloperoxidase (MPO) Activity Measurement

Myeloperoxidase activity in colon tissue homogenates was assessed following the method outlined by Bradley et al. [89] with certain modifications. The measurement of MPO activity (Abcam, Cambridge, UK) was expressed in units per gram of tissue (u/gm tissue).

### 2.16. Measurement of Proinflammatory Cytokines and Neurotransmitters

Proinflammatory cytokines, including TNF, IL-6, IL-B, and IL-10, were detected in both serum and tissue homogenate samples from the colon, intestine, and hippocampus according to the manufacturer’s instructions (MyBioSource, San Diego, CA, USA). LPS levels were measured exclusively in the serum using an ELISA DAS LPS kit (Ingenasa, Hermanos García Noblejas, Madrid, Spain). Acetylcholine, GABA, dopamine, 5-HT, and BDNF levels were specifically assessed in the hippocampus according to the manufacturer’s instructions (MyBioSource, San Diego, CA, USA).

### 2.17. Detection of Oxidative Stress Biomarkers in Serum and Tissues

The oxidative stress biomarker malondialdehyde (MDA) was measured in the hippocampus, intestine, and colon following the methodologies described by Buege and Aust, Belguendouz et al., Levine et al., and Anwar et al. [90,91,92,93,94] and according to the manufacturer’s instructions (Cayman Chemical Company, Ann Arbor, MI, USA). Enzymes such as superoxide dismutase (SOD) (Cayman Chemical Company, Ann Arbor, MI, USA), nitric oxide (NO) etiology (Abcam, Cambridge, UK), and glutathione S-transferase (GST) (Sigma-Aldrich, St. Louis, MO, USA) were also detected in the hippocampus, intestine, and colon according to Kakkar et al. [95], Miranda et al. [96], and Habig et al. [97], respectively, to assess the degree of damage and alleviation among tissues.

### 2.18. Statistical Analysis

The data are presented as the mean ± standard error of the mean (MSE) for the five groups. Statistical differences among the five groups were assessed using one-way analysis of variance (ANOVA) followed by Dunnett’s test. Comparisons between groups were performed using ANOVA with a multiple-comparisons post hoc test for normally distributed quantitative variables. For non-normally distributed quantitative variables, we utilized the Kruskal–Wallis test and the Mann–Whitney test. A significance level of *p* < 0.05 was considered statistically significant. The statistical packages used for the analysis included SPSS version 18, GraphPad (version 9.0), and Excel (version 2402).

## 3. Results

### 3.1. Construction of Ternary Phase Diagrams

**Figure 1:**
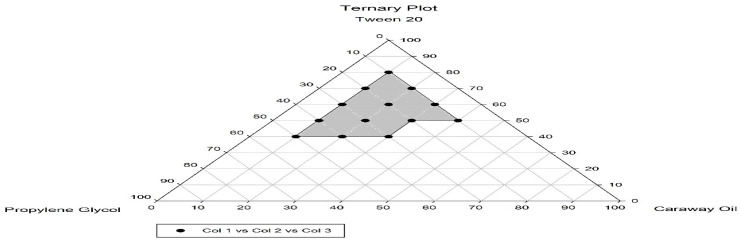
Ternary phase diagram illustrating the optimal ratios of Caraway oil, Tween 20, and propylene glycol (PG) for nanoemulsion formulation. The dots inside the shaded area indicate specific compositions that successfully form stable, translucent, or transparent nanoemulsions upon dilution with water, highlighting regions suitable for potential pharmaceutical or therapeutic applications.

The ability of the formulations to self-emulsify upon dilution along with gentle agitation was tested. As illustrated by the results of the ternary phase diagram in Figure 1, increasing the oil concentration by more than 30% led to a decrease in the self-nanoemulsifying ability of the system. Additionally, the Tween 20 concentration should be ≥40% to maintain the self-nanoemulsifying properties of the system. The rounded points represent all the self-emulsifying formulations that could emulsify spontaneously in seconds and be infinitely diluted by water.

### 3.2. Particle Size Analysis and Zeta Potential of L-MKG/SNESNS and Licorice-SNESNS

Hence, a formula composed of 30% Caraway oil, 60% Tween 20, and 10% PG was selected for drug loading because it contained a compromised surfactant/cosurfactant ratio and exhibited reasonable emulsification power upon dilution since it was crucial to adjust the Tween 20 concentration to be high enough to ensure the self-emulsification of Caraway oil.

**Figure 2:**
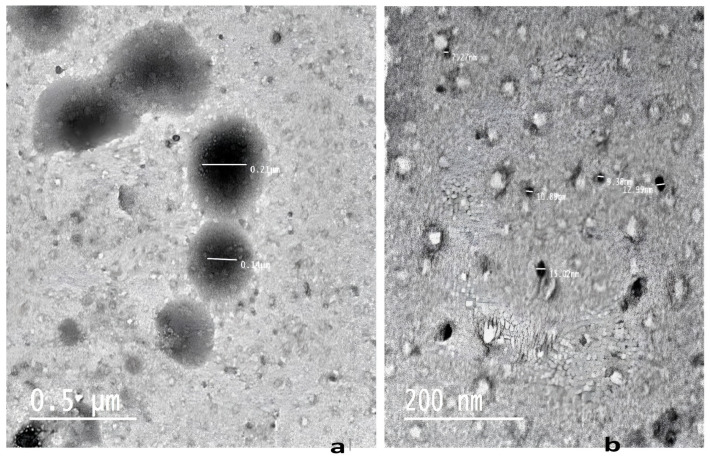
Transmission Electron Microscopy (TEM) micrographs of the lyophilized kefir-loaded licorice-SNESNS formulation. The images reveal the distinct morphology of the formulation, with black particles representing the nanosuspension (**a**), approximately 230–300 nm in size, formed after dilution with water. Additionally, smaller black droplets (**b**), measuring approximately 7–15 nm, correspond to the nanoemulsion phase. These observations confirm the successful formation of a dual nanosystem, crucial for enhancing the bioavailability and stability of the bioactive compounds.

The Z-average of the prepared licorice-SNESNS was 435.5 ± 136 nm, while that of the L-MKG/SNESNS was 435.43 ± 87.96. The PDI ranged between 0.45 and 0.48, indicating the heterogeneity of the particle size distribution. As depicted in Table 1 and Figure 2, the particle size distributions of Licorice-SNESNS and L-MKG/SNESNS showed two size peaks with different intensities. For the licorice-SNESNS formulation, the peak with an average particle size of 9.31 ± 0.27 (intensity of 46%) corresponds to the nanoemulsion globules formed. This peak shifted to a size of 114.6 ± 24.7 when licorice-SNESNS was loaded on lyophilized milk fermented by the kefir grain as shown in Table 1. At the same time, the nanosuspension particles ranged in size between 202.93 ± 31.32 and 422.1 ± 96.93 nm for the licorice-SNESNS and L-MKG/SNESNS, respectively. Upon lyophilization, the size of the droplets increased due to the increased adherence/adsorption of liquid droplets with probiotics of milk kefir grain [42]. Figure for particle size distribution and zeta potential analysis of licorice-SNESNS and lyophilized Kefir-loaded licorice-SNESNS formulations can be found in the Supplementary File S1.

The zeta potentials of the licorice-SNESNS and L-MKG/SNESNS were −6.13 ± 1.02 and −18.1 ± 0.61 mV, respectively. The increase in the negative charge of L-MKG/SNESNS is due to the presence of probiotic microorganisms such as acetic acid bacteria [98,99].

### 3.3. Morphological Analysis

TEM micrographs are shown in Figure 2. The L-MKG/SNESNS formulation was able to form a double nanosystem (nanoemulsion and nanosuspension).

### 3.4. Assessment of Body Weight and Disease Severity

**Figure 3:**
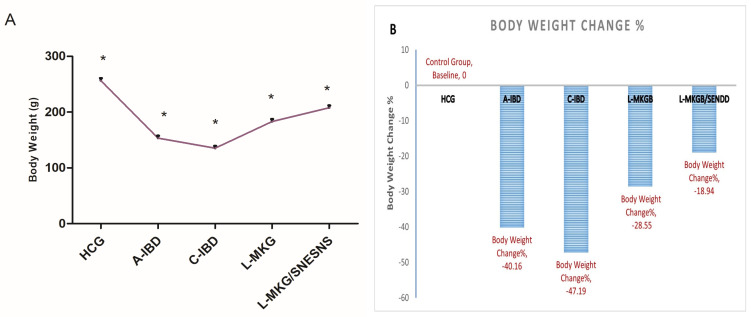

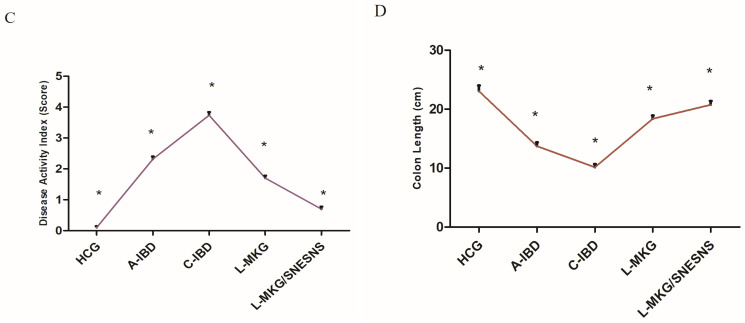
Effect of formulated L-MKG administration on disease severity. This figure illustrates the impact of lyophilized milk kefir (L-MKG) and its nanoformulated form (L-MKG/SNESNS) on disease severity in experimental models of inflammatory bowel disease (IBD). Panel (**A**) shows the changes in body weight, with measurements taken before the initiation of treatment and at the conclusion of the experimental period. Panel (**B**) shows the percentage of bodyweight change compared to the control group. The % percentage of body weight change highlights the differences in weight changes among treated and induced groups when compared to the control group, facilitating a more effective evaluation of the impact of administrated interventions. Panel (**C**) presents the disease activity index (DAI) scores, which reflect disease severity based on weight loss, stool consistency, and rectal bleeding. Panel (**D**) displays the colonic length, serving as an indicator of colonic inflammation and damage. Values are expressed as Mean ± MSE (n = 6 rats per group). Groups marked with the same asterisk indicate significance (* *p* < 0.05). Groups abbreviations: HCG (Healthy Control Group), A-IBD (Acute Inflammatory Bowel Disease), C-IBD (Chronic Inflammatory Bowel Disease), L-MKG (lyophilized milk kefir), and L-MKG/SNESNS (lyophilized milk kefir as solid carriers loaded with self-nanoemulsifying self-nanosuspension).

To investigate the impact of administering milk kefir grain beverages, whether in lyophilized or L-MKG/SNESNS formulations, on both acute and chronic IBD models, daily administration for 10 and 16 days led to notable effects. Both models were associated with reduced colon length, increased rectal bleeding, an elevated disease activity index (DAI), and altered stool consistency. These changes were more pronounced in the chronic IBD (C-IBD) group than in the acute (A-IBD) group compared to those in the Healthy Control Group, as depicted in Figure 3.

In contrast, the daily administration of LMKG/SNESNS significantly mitigated all the above-mentioned drawbacks associated with the induction of chronic IBD and almost restored the levels to those observed in the control group (*p* < 0.05). Furthermore, the administration of L-MKG alone significantly improved the colon length, DAI, and degree of weight loss. However, the administration of L-MKG/SNESNS resulted in even greater improvements, as illustrated in Figure 3. Illustration of ANOVA statistical findings conducted using SPSS, along with individual mean values for each group can be found in the Supplementary File S1, Figures S1–S3).

### 3.5. Effect on Luminal Bacterial Concentration

Table 2 provides a quantitative analysis of luminal bacterial concentrations under different experimental conditions. The counts are expressed in colony-forming units per milliliter (cfu/mL). Symbols such as +, ++, and +++ denote varying levels of bacterial presence: + indicates low, ++ indicates moderate, and +++ indicates high bacterial counts. Conversely, “-” indicates no detectable bacterial count. Specifically, beneficial bacteria including *E. coli*, *Streptococcus mitis*, *Lactobacillus casei*, and *Bacteroides fragilis* showed restored counts like the control group following treatment with L-MKG/SNESNS. In contrast, harmful strains such as Salmonella typhi, Bifidobacterium dentium, Campylobacter fetus, Bacteroides vulgatus, Klebsiella aerogenes, and Enterobacter cloacae demonstrated reduced counts after L-MKG/SNESNS treatment compared to control and L-MKG conditions.

As depicted in Table 2, L-MKG/SNESNS had a positive effect on the following bacterial strains: *E. coli*, *Streptococcus mitis*, *Lactobacillus casei*, and *Bacteroides fragilis*. The L-MKG/SNESNS-treated rats showed the restoration of normal counts of beneficial bacteria, which were similar to those of the control group. Conversely, L-MKG/SNESNS markedly decreased the count of harmful bacterial strains such as *Salmonella typhi*, *Bifidobacterium dentium*, *Campylobacter fetus*, *Bacteroides vulgatus*, *Klebsiella aerogenes*, and *Enterobacter cloacae*. The effect of L-MKG/SNESNS appeared more pronounced than that of L-MKG, potentially due to the presence of licorice extract. Previous studies have shown licorice extract to have anti-inflammatory, antioxidative stress, and immunomodulatory effects, as well as an impact on microbiota homeostasis [100].

### 3.6. Impact of Gut–Brain Disorders on Spatial Memory Dysfunctions and Anxiety-like Behaviors in an IBD-like Model Treated with L-MKG/SNESNS

**Figure 4:**
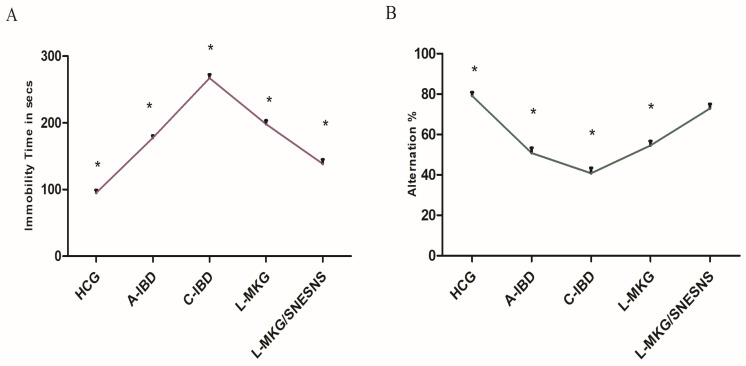
Impact of formulated L-MKG on depression, anxiety-like behavior, and spatial memory dysfunction in an IBD-DSS rat model. Panels show (**A**) Tail Suspension Test (TST) and (**B**) Y-maze represented as spontaneous alternation percentage (SAP). Values are expressed as Mean ± MSE (n = 6 rats per group). Groups marked with the same asterisk indicate significance (* *p* < 0.05). Groups abbreviations: HCG (Healthy Control Group), A-IBD (Acute Inflammatory Bowel Disease), C-IBD (Chronic Inflammatory Bowel Disease), L-MKG (lyophilized milk kefir), and L-MKG/SNESNS (lyophilized milk kefir as solid carriers loaded with self-nanoemulsifying self-nanosuspension).

The induction of both acute and chronic IBD models reduced both spatial and cognitive memory dysfunctions significantly compared to those in the control group (*p* < 0.05, Figure 4). However, the decrease in memory recognition in chronic-IBD animals was significantly alleviated by the administration of both L-MKG and L-MKG/SNESNS compared to that in the control group and C-IBD-like model, with a more substantial improvement observed upon daily administration of L-MKG/SNESNS (*p* < 0.05), as illustrated in Figure 4. Additionally, illustration of ANOVA statistical findings conducted using SPSS, along with individual mean values for each group can be found in the Supplementary File S1, Figures S4 and S5.

Additionally, a Tail Suspension Test (TST) was conducted to assess whether the IBD model induced anxiety and depression-like behaviors in both the acute and chronic groups. The results revealed that both the acute and chronic IBD-induced models displayed poor performance in the TST, especially in the chronically induced groups, compared with the control groups (*p* < 0.05). Conversely, a more pronounced reduction in immobility time was observed in both groups 4 and 5, with a greater decrease in immobility time among the group of rats administered our formulated L-MKG/SNESNS than among the control groups, as illustrated in Figure 4. Illustration of ANOVA statistical findings conducted using SPSS, along with individual mean values for each group can be found in the Supplementary File S1, Figures S4 and S5.

### 3.7. Effect on Neurotransmitter Levels and Neurotrophic Factors in an IBD Rat-like Model

**Figure 5:**
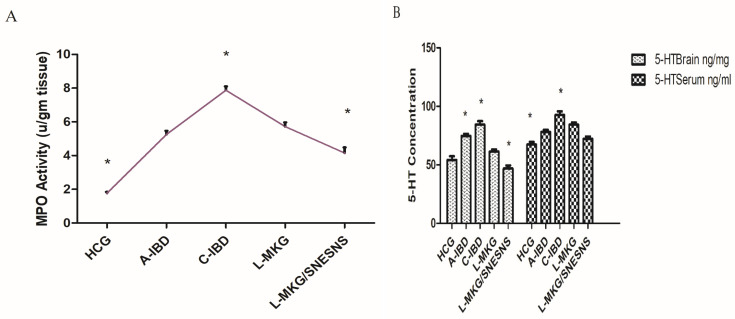
Effect of formulated L-MKG administration on colon MPO activity and 5-HT levels in the serum and brain in an IBD-DSS induced rat model. Panels show (**A**) MPO activity and (**B**) 5-HT levels. Values are expressed as Mean ± MSE (n = 6 rats per group). Groups marked with the same asterisk indicate significance (* *p* < 0.05). Groups abbreviations: HCG (Healthy Control Group), A-IBD (Acute Inflammatory Bowel Disease), C-IBD (Chronic Inflammatory Bowel Disease), L-MKG (lyophilized milk kefir), and L-MKG/SNESNS (lyophilized milk kefir as solid carriers loaded with self-nanoemulsifying self-nanosuspension).

Triggered myeloperoxidase (MPO) activity was utilized to assess the extent of neutrophil granulocyte infiltration within affected tissues, specifically colon tissue. The results indicated a significant increase in MPO activity in the IBD-like model group compared with the control group, as illustrated in Figure 5. Conversely, a notable reduction in MPO activity was observed in both treated groups (groups 4 and 5), with a more efficient reduction noted in the group of IBD rats receiving L-MKG/SNESNS (*p* < 0.05).

**Figure 6:**
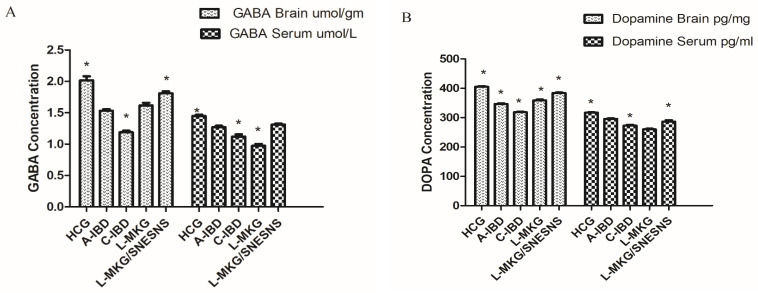

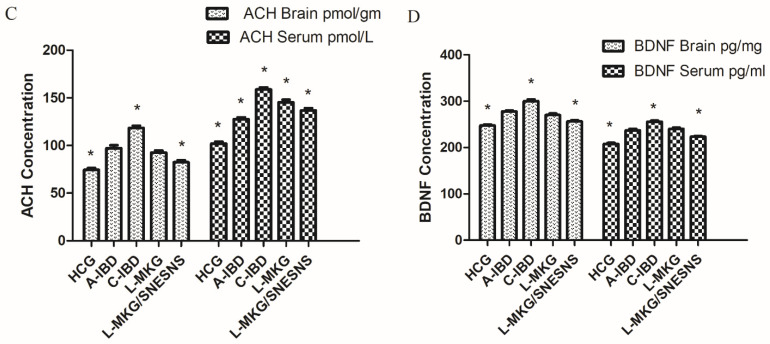
Effect of formulated L-MKG administration on neurotransmitter and neurotrophic factor levels in the serum and brain in an IBD-DSS induced rat model. Panels show (**A**) GABA, (**B**) DOPA, (**C**) ACH, and (**D**) BDNF levels. Values are expressed as Mean ± MSE (n = 6 rats per group). Groups marked with the same asterisk indicate significance (* *p* < 0.05). Groups abbreviations: HCG (Healthy Control Group), A-IBD (Acute Inflammatory Bowel Disease), C-IBD (Chronic Inflammatory Bowel Disease), L-MKG (lyophilized milk kefir), and L-MKG/SNESNS (lyophilized milk kefir as solid carriers loaded with self-nanoemulsifying self-nanosuspension).

Simultaneously, 5-hydroxytryptamine (5-HT) was measured as an indicator of direct inflammatory cell activation, revealing elevated levels in the brain and serum. Daily administration of both L-MKG and L-MKG/SNESNS successfully reversed the increase in 5-HT levels, with a more pronounced restorative effect observed in group 5 than in the control group, as depicted in Figure 6 (*p* < 0.05). The observed crosstalk in both the brain and serum implies that lyophilized milk kefir formulations potentially influence gut–brain axis disorders in IBD-like rat models. Given the crucial role of 5-HT in neurotransmission, including its involvement in mood regulation, anxiety, sleep, appetite, and gastrointestinal function, this led us to further assess the levels of other neurotransmitters. By examining these additional neurochemical changes, we aim to gain a comprehensive understanding of the treatment’s impact on the gut–brain axis compared to the control and C-IBD-like model. Illustration of ANOVA statistical findings conducted using SPSS, along with individual mean values for each group can be found in the Supplementary File S1, Figures S6 and S7.

Changes in brain levels of GABA, dopamine (DA), acetylcholine (Ach), and brain-derived neurotrophic factor (BDNF), which are markers of anxiety, depression, and behavioral dysfunctions, were observed after DSS induction of the IBD-like condition. As depicted in Figure 6, compared to those in the Healthy Control Group (HCG), various neurotransmitters, such as GABA and dopamine, exhibited reduced levels in both the acute and chronic IBD-like models (*p* < 0.05). Conversely, the levels of ACH and BDNF in both the brain and serum were greater in both the acute and chronic IBD groups than in the control group.

Upon the administration of both L-MKG and L-MKG/SNESNS, the levels of GABA, DA, Ach, and BDNF were restored compared with those in the normal control group (*p* < 0.05) in both the acute and chronic IBD-like models. A more effective restoration effect was observed in L-MKG/SNESNS group 5, as illustrated in Figure 6. This suggests that the lyophilized milk kefir grain, especially the SNESNS formulation, has a pronounced positive impact on restoring neurotransmitter levels and neurotrophic factors in the IBD rat-like model. Illustration of ANOVA statistical findings conducted using SPSS, along with individual mean values for each group can be found in the Supplementary File S1, Figures S8–S11.

### 3.8. The Alleviating Effect of Formulated Lyophilized Milk Kefir Grain on Triggered Proinflammatory Cytokines and Intestinal Permeability in an IBD-like Model

**Figure 7:**
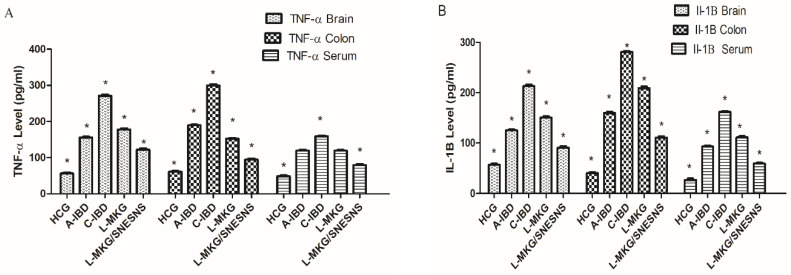

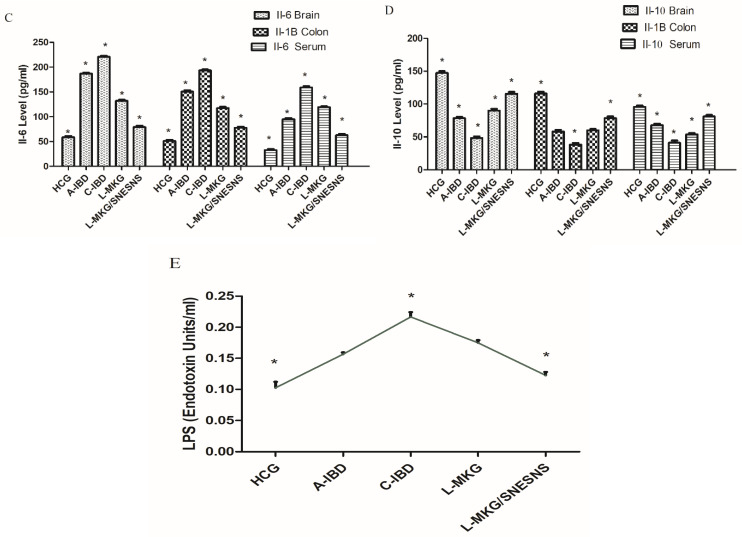
Effect of formulated L-MKG administration on triggered LPS serum levels, proinflammatory cytokines, and intestinal permeability in brain, colon, and serum in IBD-DSS induced rat model. (**A**) TNF-α, (**B**) IL-B, (**C**) IL6, (**D**) IL-10, and (**E**) LPS serum level. Values are expressed as Mean ± MSE (n = 6 rats per group). Groups marked with the same asterisk indicate significance (* *p* < 0.05). Groups abbreviations: HCG (Healthy Control Group), A-IBD (Acute Inflammatory Bowel Disease), C-IBD (Chronic Inflammatory Bowel Disease), L-MKG (lyophilized milk kefir), and L-MKG/SNESNS (lyophilized milk kefir as solid carriers loaded with self-nanoemulsifying self-nanosuspension).

Proinflammatory cascades, including the levels of TNF-α, IL-6, IL-1B, and IL-10, were assessed in the brain, serum, and colon, while lipopolysaccharide (LPS) levels were measured in the serum, as shown in Figure 7 This evaluation highlights the inflammatory response in different tissues and the systemic presence of LPS, reflecting the inflammatory status and its potential impact on the gut–brain axis. In the IBD-like model, the serum levels of TNF-α, IL-6, and IL-1B were significantly elevated in both the acute and chronic stages compared to those in the control group (*p* < 0.05). Additionally, compared with those in the control group, LPS serum levels were increased, indicating microbiota dysbiosis, gut–brain microbiota disorders, and intestinal inflammation (*p* < 0.05). These results collectively highlighted damage and injury to the intestinal barrier in IBD-like model rats.

However, daily administration of L-MKG in group 4 and L-MKG/SNESNS in group 5 reversed this damage by modulating abnormal levels of TNF-α, IL-6, IL-1B, and IL-10 in the isolated brain, intestine, and colon tissues, as well as LPS serum levels, compared with those in the healthy control group (*p* < 0.05). This suggests the efficiency of L-MKG, especially the SNESNS formulation, in alleviating the drawbacks associated with IBD. Illustration of ANOVA statistical findings conducted using SPSS, along with individual mean values for each group can be found in the Supplementary File S1, Figures S12–S16.

### 3.9. The Impact of L-MKG/SNESNS on Oxidative Stress and Antioxidant Markers in the Brain, Intestine, and Colon in an IBD-like Model

**Figure 8:**
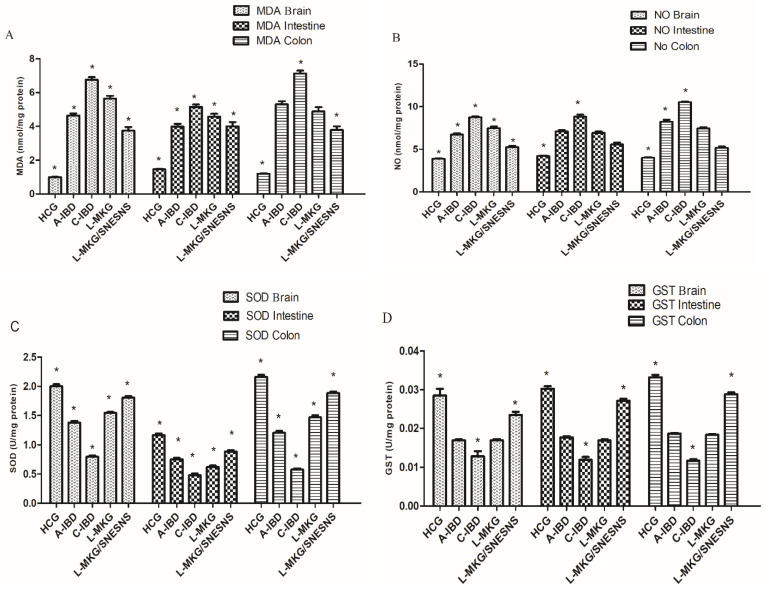
Effect of formulated L-MKG administration on oxidative stress and antioxidants in the brain, colon, and serum of DSS-induced IBD rat model. (**A**) MDA, (**B**) NO, (**C**) SOD, and (**D**) GST. Values are expressed as Mean ± MSE (n = 6 rats per group). Groups marked with the same asterisk indicate significance (* *p* < 0.05). Groups abbreviations: HCG (Healthy Control Group), A-IBD (Acute Inflammatory Bowel Disease), C-IBD (Chronic Inflammatory Bowel Disease), L-MKG (lyophilized milk kefir), and L-MKG/SNESNS (lyophilized milk kefir as solid carriers loaded with self-nanoemulsifying self-nanosuspension).

Both acute and chronic nontreated IBD-like models exhibited elevated levels of MDA, SOD, and NO in the brain, intestine, and colon, accompanied by a reduction in GST levels, as illustrated in Figure 8. In the colon, daily administration of L-MKG, whether it was loaded with SNESNS (group 5) or only L-MKG, significantly restored the MDA, SOD, and NO levels but with more pronounced effects observed following the daily administration of L-MKG/SNESNS, compared with those in the healthy control group (*p* < 0.05). This restoration was associated with an increase in GST levels, surpassing the control level, as depicted in Figure 8 Illustration of ANOVA statistical findings conducted using SPSS, along with individual mean values for each group can be found in the Supplementary File S1, Figures S17–S21.

### 3.10. L-MKG/SNESNS Mitigated Induced Inflammation, Damage, and Injuries in Histopathological Studies of the Intestine, Stomach, and Brain

**Figure 9:**
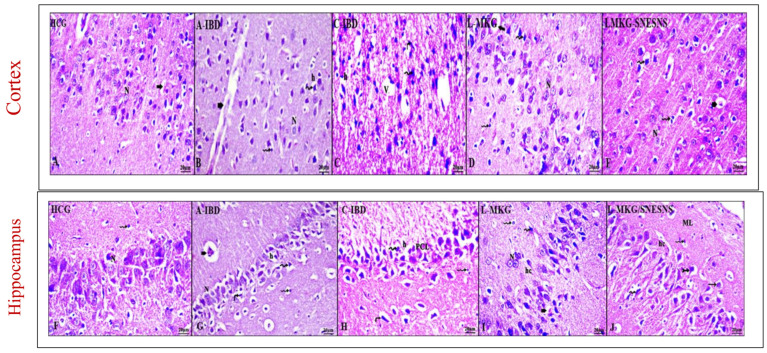
Histopathological representation of cortex and hippocampus (50×) following the administration of formulated L-MKG in IBD-DSS induced rat model. Microglial cells = Zigzag arrow, Congested blood vessel = Bifid arrow, Blood vessel = Bold arrow, Pyknotic neuron = Wavy arrow, Shrunken neuron = Curved arrow, Neuropil vacuolation = V, Granule cell layer = GCL, Pericellular haloes = h, Nuclei = N.

As shown in Figure 9, H&E staining revealed notable differences in the hippocampal and cortical brain structures among the HCG, A-IBD, and C-IBD groups. The HCG (group 1) exhibited normal histological patterns with clear long dendrites, typical neurons in a monomorphic pattern, well-formed microglia, and normal pyramidal cell structures in the cortex and hippocampus. In contrast, the A-IBD group displayed pathological brain damage, including dilated and congested blood vessels, dark nuclei with pericellular halos, and pyknotic nuclei. Hippocampal pyramidal neurons exhibited disorganization and major pyknotic nuclei. The C-IBD group showed even more severe neuropil vacuolation, fewer neurons, and darker stained nuclei, as well as more disturbances in the prefrontal cortex layers. However, the administration of both L-MKG and L-MKG/SNESNS reversed the observed brain injuries across different brain regions. Compared with those in group 3, the ability of SNESNS to modulate this damage was greater, with improvements observed in group 4 (L-MKG + C-IBD). The cerebral cortex neurons appeared in a normal standard form with scattered dilated blood vessels. The hippocampal structures showed minor normally shaped neurons and pyramidal cell bodies. The most pronounced effects were observed in the L-MKG/SNESNS treatment group 5, where intact neurons with minimal pathological changes were detected. The pia mater displayed regular attachment to the prefrontal cortex, and most cell bodies appeared normal, with a border of basophilic cytoplasm and open-face nuclei. The neuropil contained glial cells with regular blood capillaries, emphasizing the highly intact and organized nature of the hippocampal structures.

**Figure 10:**
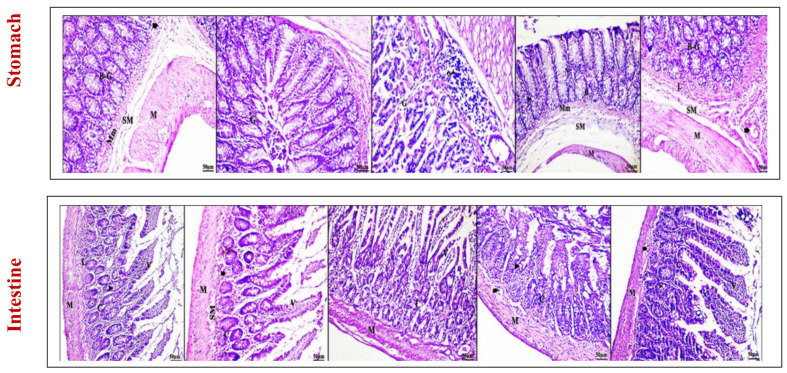
Histopathological representation of stomach and intestine (50×) following the administration of formulated L-MKG in IBD-DSS induced rat model. Mucosa (Mm), submucosa (SM), muscularis externa (M), serosa (S), villi (V), crypts region (C), congested blood vessel (bold arrow), glands (G), lamina propria (L), sunmucosa (SM) and muscularis externa (M), vertical to the fundic glands (arrows). Isthmus (I), neck (N), and base (B), dilated blood vessels (bold arrow), mononuclear cell infiltrations (wavy arrow), desquamated surface mucosal cells (curved arrow), pyknotic nuclei (zigzag arrow), cytoplasmic vacuolation in various glandular cells (bifid arrow), dilated gastric glands (D), Goblet cell metaplasia (arrowhead).

Microscopic examination of the intestinal and stomach sections was also conducted for all groups, as illustrated in Figure 10. In the HCG group, the intestine displayed no histopathological alterations, exhibiting a normal structure of the mucosa (Mm), submucosa (SM), muscularis externa (M), and serosa (S). The stomach’s light microscopic findings revealed regularly arranged, tightly packed tubular fundic glands (G), lamina propria (L), submucosa (SM), and muscularis externa (M). The luminal epithelium linings appeared thin, long, regular, and vertical to the fundic glands. Compared with that in the control group, the acute induction of IBD in group 2 resulted in intestinal damage, including degenerated epithelial cells lining the villi (v) and a deteriorated crypt region (C). Congested blood vessels (bold arrow) were also observed. The stomach sections of A-IBD patients exhibited degenerated cell structures, pyknotic nuclei (zigzag arrow), and mononuclear cells. Severe pathological damage and alterations were observed in the intestinal and stomach tissues of C-IBD patients, including a distorted mucosal architecture, hyperplasia of the columnar epithelium lining the villi, atrophied villi with major degenerative changes, sloughing of necrotic villi, and loss of villi (star). The daily administration of L-MKG and L-MKG/SNESNS resulted in significant improvements in the previously damaged intestinal and stomach tissues. Compared with the C-IBD group, the L-MKG group showed a relative rate of improvement in the intestine, with organized structures and little degree of degeneration. The villi structure and crypts of the intestinal gland appeared to be intact. Moreover, substantial improvement was detected in the stomach of the LMKG group (group 4) compared to that of group 3, with a slight normal intact structure of fundic glands (G), submucosa (SM), and muscularis (M). Compared with those in the HCG group, the microscopic structures of the stomach and intestine in the L-MKG/SNESNS group 5 were more intact. A clear lumen was observed, and the intestinal villi tended to preserve their natural shape. The stomach exhibited an intact structure with no dilated blood vessels or mononuclear cell infiltrations.

**Figure 11:**
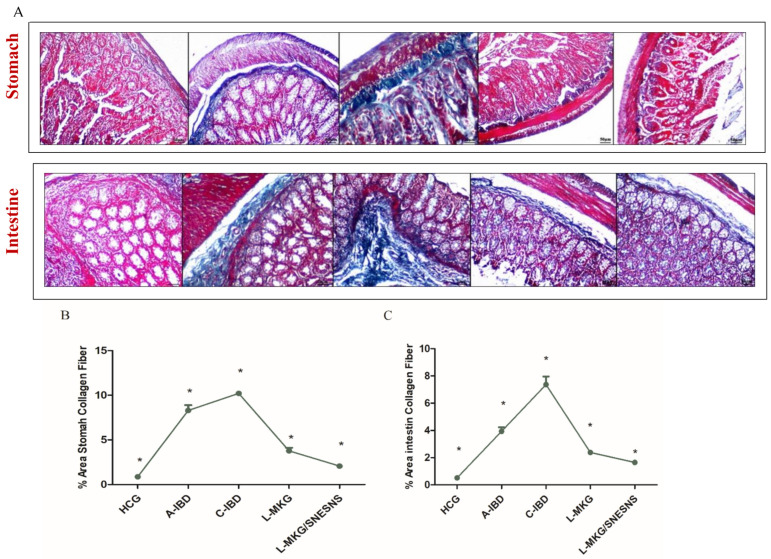
Representative tissue sections of stomach and intestine stained with Masson’s trichrome staining illustrating the effect of formulated L-MKG administration on increased deposits of collagen in IBD-DSS induced rat model. (**A**) Masson’s trichrome blue staining demonstrates the degree of collagen deposits in the stomach and intestine of IBD-DSS induced rat model (50×). (**B**) Percentage area of the degree of collagen fiber deposits in stomach. (**C**) Percentage area of the degree of collagen fiber deposits in intestine. Values are expressed as Mean ± MSE (n = 6 rats per group). Groups marked with the same asterisk indicate significance (* *p* < 0.05). Groups abbreviations: HCG (Healthy Control Group), A-IBD (Acute Inflammatory Bowel Disease), C-IBD (Chronic Inflammatory Bowel Disease), L-MKG (lyophilized milk kefir), and L-MKG/SNESNS (lyophilized milk kefir as solid carriers loaded with self-nanoemulsifying self-nanosuspension).

To assess the degree of inflammation and infiltration among all groups, Masson’s trichrome staining was used to analyze collagen production. The staining type revealed increased collagen deposition in the stomach and intestine, both under chronic and acute conditions, with more prominent deposits compared to those in the control group. The degree of collagen deposition increased as the disease progressed, as indicated by the % area content of collagen fibers in Figure 11. In contrast, compared with those in the control group, Masson’s trichrome staining revealed more normal structures in the intestine and stomach, particularly in group 5, than in group 4. This observation was further supported by the calculated % mean area of collagen fibers in both treated groups, as depicted in Figure 11.

## 4. Discussion

Homeostasis of the gut microbiota refers to maintaining a stable and balanced microbial composition within the host immune system. Persistent disruptions to this equilibrium can lead to dysbiosis, characterized by the reduced diversity and depletion of beneficial microbes [15,101,102]. Patients with IBD are prone to mental health issues such as anxiety, depression, and cognitive decline due to oxidative stress, intestinal injury, increased intestinal permeability, and gut–brain axis abnormalities. Recent evidence highlights the role of microbiota–gut–brain axis disorders in cognitive decline [6,15,103,104]. Numerous bacterial species detected in the brains of postmortem IBD patients suggest microbes may contribute to cognitive decline and neuroinflammation. An imbalance in gut microbiota can trigger inflammatory responses in the intestines, damaging the intestinal barrier. This damage may allow pathogenic bacteria to enter the systemic circulation, leading to Blood–Brain Barrier (BBB) destruction, cognitive decline, and neuroinflammation [102,104].

Our previous findings demonstrated the effectiveness of milk kefir grains in combating cognitive dysfunctions and the deposition of amyloid-beta plaques [22,23,24]. The use of milk kefir grain starter requires specific procedures and inoculation to activate the grains correctly. One of our study’s objectives is to develop an efficient lyophilized nanoformulation of milk kefir grains and compare it with the standard lyophilized grain. Combining kefir with a self-nanoemulsifying system (SNESNS) containing Caraway oil and licorice extract enhances the bioavailability of these bioactive compounds [105,106,107]. This increased bioavailability can lead to improved therapeutic outcomes compared to using kefir alone. Our formulation simplifies the use of kefir by providing it in a lyophilized powder form, making it easier to store, handle, and administer, thus offering greater convenience for patients [108]. This method eliminates the need for specific conditions and expertise required for the traditional preparation and activation of kefir grains. Lyophilization ensures the stability and consistency of the formulation, which is crucial for maintaining the therapeutic efficacy of the bioactive compounds [109]. By converting kefir into a lyophilized form combined with nanoemulsified bioactive compounds, we overcome common barriers to traditional kefir use, such as the necessity for refrigeration and daily preparation. This makes the product more accessible and user-friendly, particularly for individuals who may not have the time or resources to manage traditional kefir fermentation. While our primary aim was to showcase the enhanced efficacy and practicality of our novel formulation and the therapeutic outcomes of the lyophilized kefir grain when compared with our previous findings [22,23,24], this comparative analysis highlights the superior performance of our formulation in providing improved therapeutic effects. Our study provides strong scientific support for the use of lyophilized kefir combined with nanoemulsifying systems, establishing the benefits and potential applications of this innovative formulation in various therapeutic areas.

The conventional therapeutics for treating IBD primarily consist of several anti-inflammatory and antioxidant agents. However, their immune system side effects, including both short- and long-term disabilities, limit prolonged use [15,104,110]. Therefore, there is an urgent need for more efficient, affordable, and alternative treatments. One promising approach is targeting natural ingredients through nanodrug-delivery systems, which show the potential to enhance IBD treatment efficiency. The intestinal microbiota plays a crucial role in various diseases by directly interacting with the brain, immune system, and disease-related pathways involving the host’s endocrine, nervous, and immune systems [15,104,110]. To evaluate the efficiency of probiotics in treating IBD and related gut–brain microbiota disorders that lead to cognitive deterioration, we focused on encapsulating probiotics in nanosystems to increase their stability, bioavailability, and effectiveness [110]. Kefir, derived from grains, possesses probiotic characteristics [2,18,111,112]. The unique symbiotic mixture of microorganisms in kefir includes various strains that promote health by maintaining intestinal microbial balance and exerting anti-inflammatory and antioxidant effects [2,111,112].

In this study, we emphasize the impact of IBD on the brain and the gut–brain–microbiota axis disorders. We examined IBD’s effects on brain damage, neuroinflammation, cognitive decline, and behavioral changes. Additionally, we assessed the efficacy of daily administration of L-MKG/SNESNS in alleviating IBD and its associated cognitive decline and spatial memory damage. We generated various IBD animal models, including acute and chronic models, using DSS to induce intestinal inflammation resembling that in humans. The acute and chronic IBD colitis models were characterized by weight loss, colon length, DAI, oxidative stress, and inflammatory markers. Gut inflammation results in severe endothelial cell dysfunction, allowing proinflammatory mediators to penetrate the brain via the BBB, associated with increased immune factors in IBD patients’ serum [113].

DSS has been used to induce IBD-like colitis models in rodents, increasing intestinal wall permeability, compromising mucosal barrier integrity, and enhancing inflammatory cytokine infiltration. The DSS-induced rat model highlighted the significant role of the gut microbiota in IBD etiopathogenesis and its associated microbiota–gut–brain axis disorder, along with cognitive dysfunctions and behavioral changes [5,18,114]. Our results showed that DSS-challenged rats had increased DAI scores in both acute and chronic models, with significant intestinal and stomach lesions, increased collagen deposition, and shorter colon length. Histopathological analysis indicated mucosal structure injuries, especially with daily administration for more than 16 days, suggesting IBD disorders induced by DSS, with inflammatory cells infiltrating the intestines. MPO activity, reflecting inflammation, infiltration, and mucosal damage, was significantly increased in colon tissue homogenates, indicating neutrophilic colonic infiltration. This infiltration was associated with marked alterations in microbiota composition [5,114,115]. Our previous findings showed that the daily administration of milk kefir grain as a beverage was effective against Alzheimer’s disease and neuroinflammation due to its potent antioxidant, antiapoptotic, and anti-inflammatory effects and its ability to trigger neurotrophic factors [22,23,24,116]. In this study, to enhance efficiency, stability, and bioavailability, the L-MKG beverage was lyophilized and/or prepared in SNESNS. Daily administration of milk kefir grains restored and modulated the damage induced by IBD. Both L-MKG and/or L-MKG/SNESNS restored histopathological findings of the stomach and intestine, with L-MKG/SNESNS showing greater efficacy [2,18,112]. Additionally, decreased DAI, colon length, body weight, fecal composition, and MPO activity were observed. These findings suggest that L-MKG and/or L-MKG/SNESNS are promising treatments for DSS-induced IBD-like colitis.

Our results revealed that both acute and chronic IBD are associated with impaired memory functions and behavioral changes, as evaluated by the Y-maze and TST, due to brain injury, gut–brain axis dysfunction, and neuroinflammation. Consistent with our findings, DSS-induced models are also linked to behavior disorders and spatial memory dysfunctions [9,117,118]. These behavioral defects were restored following early administration of L-MKG/SNESNS, indicating the efficacy of L-MKG in both formulations for alleviating IBD and gut–brain microbiota disorders [2,15,18,119]. During the fermentation process of milk kefir grain starters, bacteria release various beneficial compounds. Lactic acid bacteria, including Lactobacillus, Streptococcus, and Bifidobacteria, are the most abundant bacterial compounds responsible for a wide range of health benefits, along with exopolysaccharides [119,120,121]. However, the active constituents of kefir grains can vary based on their place of origin [121]. Most Lactobacillus species are classified as beneficial probiotic organisms and are highly abundant in kefir grains [119,121]. In this study, a decrease in fecal Lactobacillus concentration was more prominent in the chronic IBD model than in the acute model. Daily administration of L-MKG/SNESNS and L-MKG restored these modifications, suggesting that Lactobacillus metabolites positively affected memory functions and restored gut homeostasis [2,9,18]. Daily Lactobacillus consumption is suggested to benefit spatial memory by increasing GABA expression in the hippocampus and positively affecting the vagus nerve, which is the main gut–brain axis responsible for shifts in brain microbiota disorders [2,111].

Short-chain fatty acids (SCFAs), produced by Lactobacillus-type probiotics through the metabolism of intestinal flora, repair intestinal damage, promote the colonization of beneficial bacteria, and improve cognitive functions while alleviating anxiety [2,115,117]. SCFAs also regulate acquired and adaptive immunity and increase intestinal rigidity. Milk kefir grain has the potential to mediate a high diversity of microbiota through continuous SCFA colonization, playing a crucial role in mitigating cognitive dysfunctions, inflammation, immunological disorders, anxiety, and depression [2,115,117]. Kefir has been shown to increase the diversity of intestinal microbiota through SCFA colonization. DSS-induced colitis and IBD models suffer from low SCFA intestinal colonization, including metabolites like acetate, butyrate, and propionate. The efficacy of kefir against IBD, spatial memory dysfunctions, and behavior disorders may be linked to increased SCFA production and elevated levels of acetate, butyrate, and propionate in the feces of affected models [2,18].

Neurotransmitters like acetylcholine, dopamine, GABA, and 5-HT are produced in the central nervous system (CNS) and play crucial roles in functions such as intestinal tissue repair, permeability, cognitive functions, behavior, emotional processing, and spatial memory. Recent studies indicate that the gut microbiota can influence these neurotransmitters, affecting cognitive abilities and behavior. Modulating the microbiota–gut–brain axis may regulate brain injury, anxiety, and cognitive function. Cognitive dysfunctions, anxiety, and behavioral changes in acute and chronic DSS rats may be associated with disturbances in neurotransmitter levels, especially 5-HT, GABA, dopamine, and acetylcholine [5,117,122]. The concentration of 5-HT, involved in emotional control, appetite regulation, and pain modulation, was high in both acute and chronic IBD models, consistent with previous studies [118,123,124,125]. The overproduction of 5-HT is associated with anxiety, depression, and inflammation, triggered by altered intestinal microbiota that activate immune cells and inflammation [125,126]. GABA, the most abundant inhibitory neurotransmitter, is suppressed in IBD, leading to cognitive decline, anxiety, insomnia, restlessness, and behavioral changes [117,127]. Additionally, decreased dopamine levels, crucial for spatial memory and behavior regulation and acting as an anti-inflammatory neuroactive substance, were observed in IBD models [117]. Conversely, increased levels of BDNF and acetylcholine were found in IBD model rats, likely related to increased inflammation and elevated lipopolysaccharide (LPS) serum levels [118,128,129,130]. LPS triggers inflammation, and its elevated levels in IBD models may result from complex intestinal microflora, leading to inflammatory cytokine release and brain damage [114,128]. Consistent with these findings, daily administration of milk kefir and probiotics restored neurotransmitter levels to normal in colitis and IBD models [15,131,132].

The intestinal barrier plays a crucial role in containing the gut microbiota and preventing its entry into systemic circulation. Dysbiosis can profoundly affect both innate and adaptive immunity, triggering intestinal inflammation and disrupting the intestinal barrier’s integrity [104,110,133]. This disruption can lead to age-related diseases, severe systemic inflammation, microgliosis, and the activation of the M1 phenotype, ultimately resulting in neuroinflammation [104,110,133,134,135,136]. Proinflammatory cytokines are central to cellular interactions within the intestine, influencing immune regulation based on inflammation severity and tissue penetration [104,110,133,134,135,137]. In our study, IBD-induced proinflammatory cytokines disrupted Blood–Brain Barrier (BBB) integrity, increasing BBB permeability and contributing to neuroinflammation [104,110,118,122,133]. These cytokines, acting as critical biomarkers, are released in the intestinal mucosa and systemic circulation. Elevated levels of TNF-α, IL-6, and IL-1β in the colon, brain, and serum, along with downregulated IL-10, were observed in acute and chronic IBD-like models in rats [104,110,118,122,133]. These alterations in cytokine levels are strongly linked to IBD pathogenesis and brain injury, with proinflammatory cytokines potentially entering the brain, disrupting homeostasis, and causing microgliosis, cognitive decline, and neuroinflammation [138,139,140].

Dysbiosis of the gut microbiota triggers local inflammatory responses, disrupts intestinal barrier integrity, and induces systemic inflammation that can penetrate the BBB, leading to cognitive decline. Our results indicate that daily oral administration of probiotics and L-MKG in both formulations has significant potential as an alternative treatment for IBD and its associated gut–brain axis disorders. This is due to their ability to reestablish gut microbiota homeostasis, restore gut barrier integrity, maintain normal immune status, act as a barrier against pathogens, alleviate systemic inflammation, and consequently mitigate cognitive decline [15,18,104,127,132]. There is cross-talk between proinflammatory cytokine overproduction and oxidative stress in IBD. Increased oxidative stress, closely associated with mucosal inflammation, may contribute to brain disorders, cognitive decline, and behavioral changes. It plays a significant role in the pathogenesis of intestinal damage in IBD, resulting from increased free radicals and the depletion of antioxidant defenses [4,122,141]. In our study, IBD induction in a rat model was accompanied by increased NO and MDA levels and decreased SOD and GST levels in various tissues and serum. Colitis and IBD exhibit manifestations in the brain, intestine, stomach, and heart due to severe inflammation. Elevated NO and MDA levels resulted in severe neuronal damage, increasing oxidative stress and weakening defense mechanisms. Disruption of the balance between oxidant and antioxidant systems leads to severe systemic disorders [4,122,141]. Daily administration of LMKG significantly reduced NO and MDA levels and increased SOD and GST activities due to its antioxidant and anti-inflammatory properties and beneficial constituents produced during fermentation [18,142,143].

The findings of this study underscore the multifaceted role of the microbiota–gut–brain axis in IBD and associated cognitive decline. Our data suggest that the administration of lyophilized milk kefir grains (L-MKGs), especially in the form of a self-nanoemulsifying system, has the potential to mitigate the adverse effects of IBD on both gastrointestinal and neurological health. The observed decrease in oxidative stress markers and improvement in histopathological parameters highlight the antioxidative and anti-inflammatory properties of the treatment. These outcomes not only provide insights into the pathophysiological link between gut microbiota disturbances and neuroinflammation but also support the therapeutic potential of probiotics and nanotechnology-based delivery systems in managing IBD and related cognitive disorders. The study’s approach to maintaining probiotics in a nanoformula for enhanced stability and bioavailability could serve as an outline for developing targeted therapies that address the gut–brain axis. Further research should explore the mechanistic pathways involved, such as specific microbial strains and metabolites that confer neuroprotection, and investigate the long-term effects of such treatments in clinical settings.

## 5. Focused Limitations of the Study

Despite the promising findings, our study has a notable limitation in that we only included male rats in our experiments. This gender-specific selection may not fully capture potential differences in response to the treatment between male and female subjects. Given that sex differences in the microbiota–gut–brain axis, cognitive function, and response to therapeutics are well-documented, excluding female rats limits the generalizability of our findings to both sexes. Therefore, future studies should incorporate both male and female subjects to provide a more comprehensive understanding of the effects of lyophilized kefir and SNESNS. Additionally, the small sample size limits the generalizability of our results, underscoring the need for larger studies to confirm our findings. Furthermore, the short-term observation period does not account for the potential long-term effects of the treatment, which should be investigated in future research.

## 6. Conclusions and Future Research Perspectives

In summary, inflammatory bowel disease (IBD) and neurological disorders can significantly diminish patients’ quality of life. This underscores the imperative for increased and effective scientific research aimed at developing viable alternatives to mitigate these disorders. Within the scope of our current study, we demonstrated the potential of using lyophilized probiotics as solid carriers loaded with a self-nanoemulsifying drug-delivery system to selectively target highly inflamed intestinal mucosa. This system effectively mitigated spatial memory damage while promoting several healing factors. Additionally, the IBD-like model used in this study contributed to uncovering neurobiological alterations within the central nervous system (CNS). These findings provide valuable insights that can guide the development of alternative therapeutic options to alleviate IBD-associated disorders, particularly cognitive decline and memory dysfunctions, along with increasing patients’ quality of life.

## Figures and Tables

**Table 1:** Particle size, PDI, and ZP of licorice-SNESNS and lyophilized kefir-loaded licorice-SNESNS.

Formula	Z-average (nm)	Peak 1	Peak 2	PDI	ZP (mV)
**Licorice-SNESNS**	435.5 ± 136	202.9 ± 31	9.3 ± 0.3	0.45 ± 0.074	−6.1 ± 1.02
**Lyophilized kefir-loaded licorice-SNESNS**	435.4 ± 88	422.1 ± 97	114.6 ± 25	0.48 ± 0.067	−18.1 ± 0.61

**Table 2:** The effects of L-MKG and L-MKG/SNESNS on bacterial count in an IBD-DSS induced rat model.

Bacterial Strains	Control	Count ×10^3^ cfu	Induction 1 after 10 Days	Count ×10^3^ cfu	Induction 1 after 16 Days	Count ×10^3^ cfu	L-MKG	Count ×10^3^ cfu	L-MKG/ SNESNS	Count ×10^3^ cfu
***E. coli* (*n*)**	**+++**	5.5	+	3.8	+	2.5	++	4.7	+++	6.2
***Streptococcus mitis* (*n*)**	**++**	2.3	+	1.8	+	1.2	+	1.6	++	2.5
***Lactobacillus casei* (*n*)**	**++**	2.5	+	1.7	+	1.0	+	1.8	++	2.5
***Bacteroides fragilis* (*n*)**	**++**	3.2	+	2.5	+	1.7	+	2.5	++	3.5
***Salmonella typhi***	***-***	0.1	++	2.6	++	3.5	+	2.3	+	1.2
***Bifidobacterium dentium***	***-***	0.2	++	3.2	++	4.2	+	2.8	+	0.8
***Campylobacter fetus***	***-***	0.1	++	3.5	++	4.5	+	3.1	+	1.8
***Bacteroides vulgatus***	***-***	0.0	+	2.2	++	3.3	+	1.8	+	0.7
***Klebsiella aerogenes***	***-***	0.2	+	3.3	++	4.4	+	3.2	+	1.5
***Enterobacter cloacae***	***-***	0.0	+	2.8	++	3.8	+	1.8	+	0.5

## Data Availability

The original contributions presented in the study are included in the article and Supplementary Materials, and further inquiries can be directed to the corresponding author.

## Supplementary Materials

The following supporting information can be downloaded at: https://www.mdpi.com/article/10.3390/antiox13101205/s1. Figure S1: Illustration of ANOVA statistical findings conducted using SPSS, along with individual mean values for each group regarding body weight. Figure S2: Illustration of ANOVA statistical findings conducted using SPSS, along with individual mean values for each group regarding Disease Activity Index (DAI). Figure S3: Illustration of ANOVA statistical findings conducted using SPSS, along with individual mean values for each group regarding Colon Length. Figure S4: Illustration of ANOVA statistical findings conducted using SPSS, along with individual mean values for each group regarding Tail Suspension Test. Figure S5: Illustration of ANOVA statistical findings conducted using SPSS, along with individual mean values for each group regarding Y Maze. Figure S6: Illustration of ANOVA statistical findings conducted using SPSS, along with individual mean values for each group regarding MPO. Figure S7: Illustration of ANOVA Statistical Findings Conducted Using SPSS, along with individual mean values for each group regarding 5-HT Levels in Brain and Serum, Respectively. Figure S8: Illustration of ANOVA Statistical Findings Conducted Using SPSS, along with individual mean values for each group regarding GABA Levels in Brain and Serum, Respectively. Figure S9: Illustration of ANOVA Statistical Findings Conducted Using SPSS, along with individual mean values for each group regarding Dopamine Levels in Brain and Serum, Respectively. Figure S10: Illustration of ANOVA Statistical Findings Conducted Using SPSS, along with individual mean values for each group regarding ACH Levels in Brain and Serum, Respectively. Figure S11: Illustration of ANOVA Statistical Findings Conducted Using SPSS, along with individual mean values for each group regarding BDNF Levels in Brain and Serum, Respectively. Figure S12: Illustration of ANOVA Statistical Findings Conducted Using SPSS, along with individual mean values for each group regarding TNF-α Levels in Brain, Colon, and Serum, Respectively. Figure S13: Illustration of ANOVA Statistical Findings Conducted Using SPSS, along with individual mean values for each group regarding IL-1B Levels in Brain, Colon, and Serum, Respectively. Figure S14: Illustration of ANOVA Statistical Findings Conducted Using SPSS, along with individual mean values for each group regarding IL-6 Levels in Brain, Colon, and Serum, Respectively. Figure S15: Illustration of ANOVA Statistical Findings Conducted Using SPSS, along with individual mean values for each group regarding IL-10 Levels in Brain, Colon, and Serum, Respectively. Figure S16: Illustration of ANOVA statistical findings conducted using SPSS, along with individual mean values for each group regarding LPS. Figure S17: Illustration of ANOVA Statistical Findings Conducted Using SPSS, along with individual mean values for each group regarding MDA Levels in Brain, Intestine, and Colon, Respectively. Figure S18: Illustration of ANOVA Statistical Findings Conducted Using SPSS, along with individual mean values for each group regarding NO Levels in Brain, Intestine, and Colon, Respectively. Figure S19: Illustration of ANOVA Statistical Findings Conducted Using SPSS, along with individual mean values for each group regarding SOD Levels in Brain, Intestine, and Colon, Respectively. Figure S20: Illustration of ANOVA Statistical Findings Conducted Using SPSS, along with individual mean values for each group regarding GST Levels in Brain, Intestine, and Colon, Respectively. Figure S21: Particle size distribution and zeta potential analysis of licorice-SNESNS and lyophilized Kefir-loaded licorice-SNESNS formulations. The particle size analysis illustrates the size distribution of the nanoemulsion and nanosuspension particles, indicating the formulation’s homogeneity and stability. Zeta potential measurements provide insight into the surface charge and colloidal stability of the formulations, with higher absolute values suggesting greater stability against aggregation.
