# Supplementary material for: Impact of Lyophilized Milk Kefir-Based Self-Nanoemulsifying System on Cognitive Enhancement via the Microbiota–Gut–Brain Axis
Source: Antioxidants (Basel). 2024 Oct 7;13(10):1205. doi: 10.3390/antiox13101205 (PMC11504727; doi:10.3390/antiox13101205)
Supplement: Supplementary file 1 [file antioxidants-13-01205-s001.zip › antioxidants-3209897-supplementary.pdf]

1- Main Isolation Media Used

1. **Blood Agar (BA):** Incubated for 16-24 hours in 5-10% CO_2 at 35-37°C.
2. **MacConkey Agar (MAC):** Incubated for 16-24 hours in ambient air at 35-37°C.
3. **Cystine-Lactose-Electrolyte Deficient (CLED) Agar with Bromothymol Blue (CLED B) or Andrade's Indicator (CLED A):** Incubated for 16-24 hours in ambient air at 35-37°C.
4. **Selective Enteric Media:** Incubated in ambient air at 35-37°C for 16-24 hours:
   - **Xylose-Lysine-Deoxycholate Agar (XLD).**
   - **Brilliant Green Agar (BGA).**
   - **Thiosulfate-Citrate-Bile Salt (TCBS) Agar.**
5. **Chromogenic Media:** Incubated in ambient air at 35-37°C for 16-24 hours.

2- Colonial Appearance

1. **Blood Agar (BA):**
   - **Colonies:** 2-3 mm in diameter, low, convex, gray, smooth, or mucoid.
   - **Characteristics:** Colonies may exhibit hemolytic activity or show swarming behavior.
2. **MacConkey Agar (MAC):**
   - **Colonies:** Pink (lactose fermenting) or colorless (lactose non-fermenting).
   - **Characteristics:** Size and shape of colonies vary with individual species.
3. **Cystine-Lactose-Electrolyte Deficient (CLED) Agar with Bromothymol Blue (CLED B):**
   - **Colonies:** Yellow (lactose fermenting) or blue (lactose non-fermenting).
   - **Characteristics:** Size and shape of colonies vary with individual species.
4. **Cystine-Lactose-Electrolyte Deficient (CLED) Agar with Andrade's Indicator (CLED A):**
   - **Colonies:** Pink (lactose fermenting) or green, translucent (lactose non-fermenting).
   - **Characteristics:** Size and shape of colonies vary with individual species.
5. **Xylose-Lysine-Deoxycholate Agar (XLD):**
   - **Colonies:** Yellow (xylose, lactose, or sucrose fermenting) or pink (non-fermenting).
   - **Characteristics:** May show a black center for H_2S producers.

6. **Brilliant Green Agar (BGA):**
   - **Colonies:** Red-pink, 1-3 mm in diameter, surrounded by brilliant red zones in the agar.
7. **Thiosulfate-Citrate-Bile Salt (TCBS) Agar:**
   - **Colonies:** Yellow (sucrose fermenting) or blue-green (sucrose non-fermenting).

3- ANOVA Results and Individual Mean Values for the Observed Variables

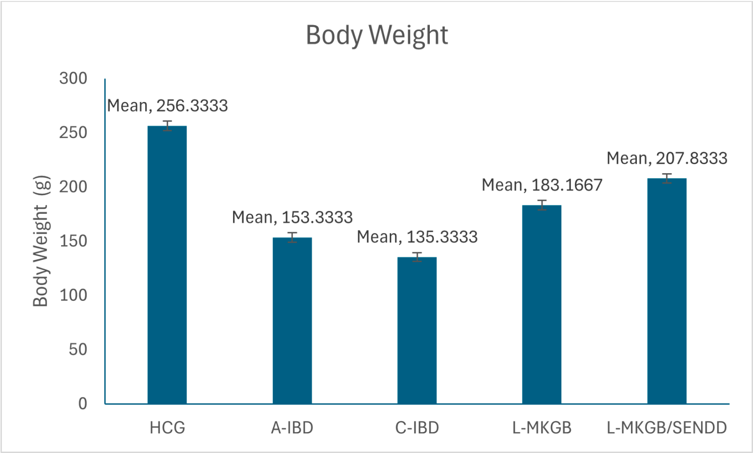

ANOVA

BW

	Sum of Squares	df	Mean Square	F	Sig.
Between Groups	54351.133	4	13587.783	121.682	.000
Within Groups	2791.667	25	111.667		
Total	57142.800	29			

Figure S1. Illustration of ANOVA statistical findings conducted using SPSS, along with individual mean values for each group regarding body weight.

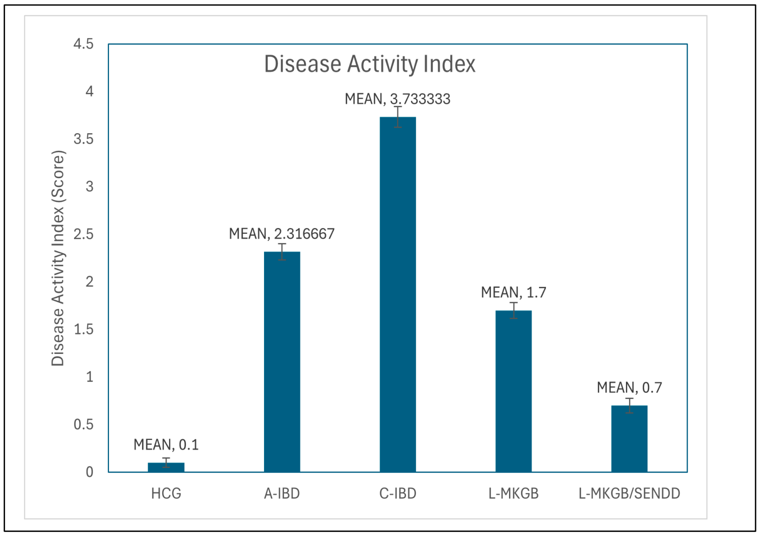

ANOVA

DAI

	Sum of Squares	df	Mean Square	F	Sig.
Between Groups	48.445	4	12.111	247.844	.000
Within Groups	1.222	25	.049		
Total	49.667	29			

Figure S2. Illustration of ANOVA statistical findings conducted using SPSS, along with individual mean values for each group regarding Disease Activity Index (DAI).

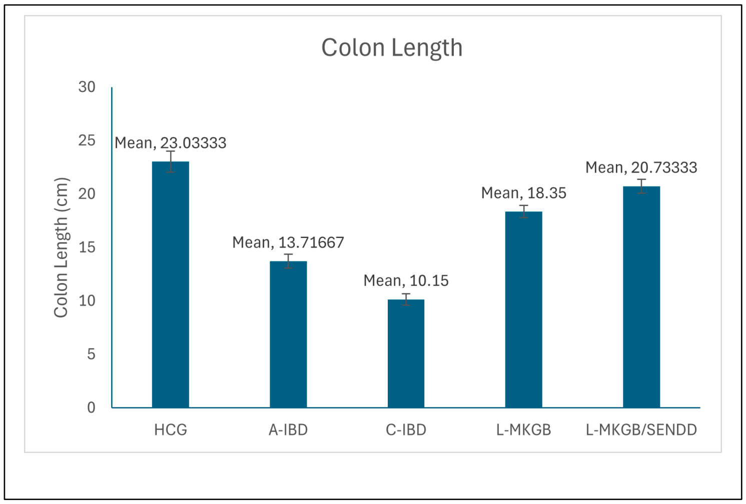

ANOVA

CL

	Sum of Squares	df	Mean Square	F	Sig.
Between Groups	658.025	4	164.506	56.754	.000
Within Groups	72.465	25	2.899		
Total	730.490	29			

Figure S3. Illustration of ANOVA statistical findings conducted using SPSS, along with individual mean values for each group regarding Colon Length.

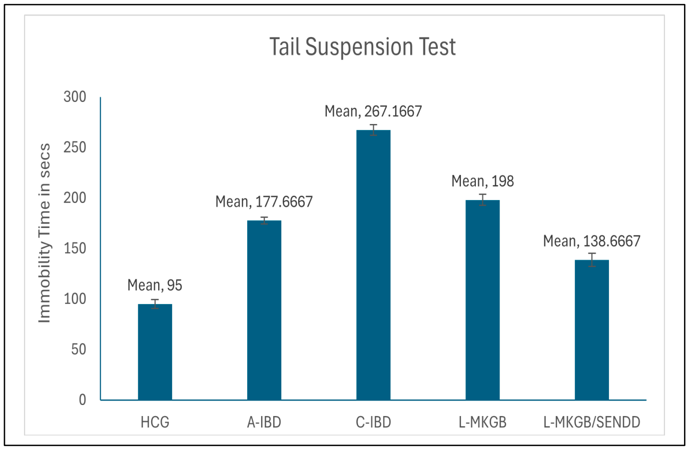

ANOVA

TST

	Sum of Squares	df	Mean Square	F	Sig.
Between Groups	100502.800	4	25125.700	132.982	.000
Within Groups	4723.500	25	188.940		
Total	105226.300	29			

Figure S4. Illustration of ANOVA statistical findings conducted using SPSS, along with individual mean values for each group regarding Tail Suspension Test.

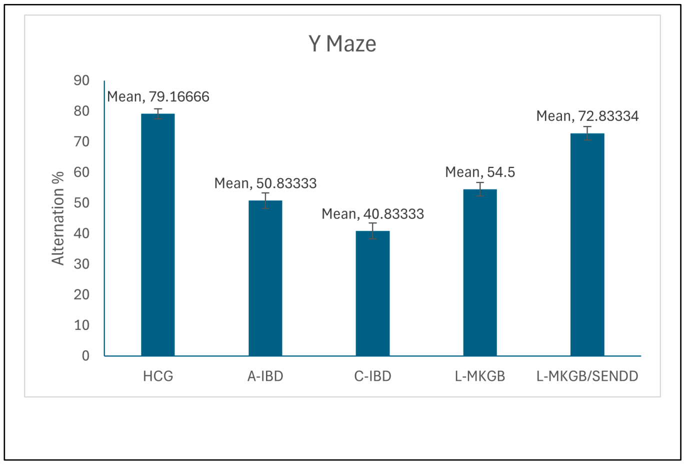

ANOVA

YMaze

	Sum of Squares	df	Mean Square	F	Sig.
Between Groups	6078.133	4	1519.533	41.076	.000
Within Groups	924.833	25	36.993		
Total	7002.967	29			

Figure S5. Illustration of ANOVA statistical findings conducted using SPSS, along with individual mean values for each group regarding Y Maze.

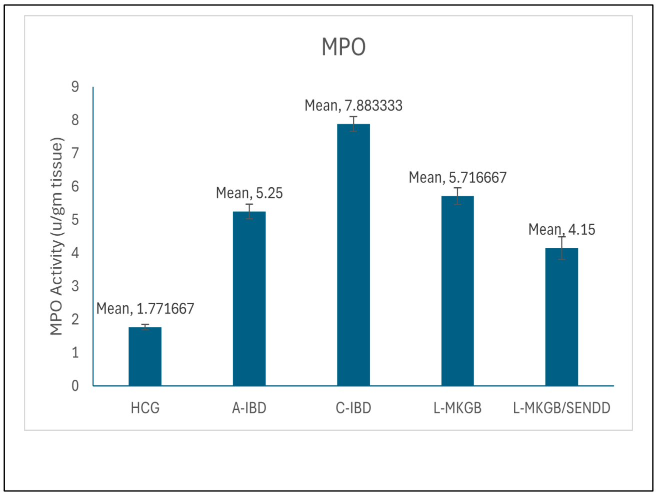

ANOVA

MPO

	Sum of Squares	df	Mean Square	F	Sig.
Between Groups	120.144	4	30.036	89.119	.000
Within Groups	8.426	25	.337		
Total	128.569	29			

Figure S6. Illustration of ANOVA statistical findings conducted using SPSS, along with individual mean values for each group regarding MPO.

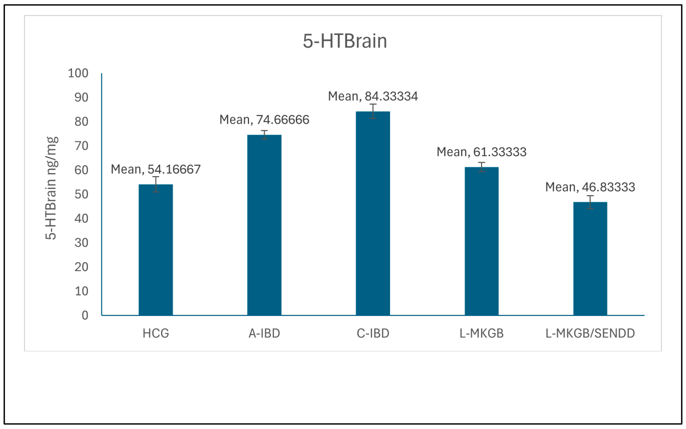

ANOVA

brainHt

	Sum of Squares	df	Mean Square	F	Sig.
Between Groups	5552.200	4	1388.050	29.821	.000
Within Groups	1163.667	25	46.547		
Total	6715.867	29			

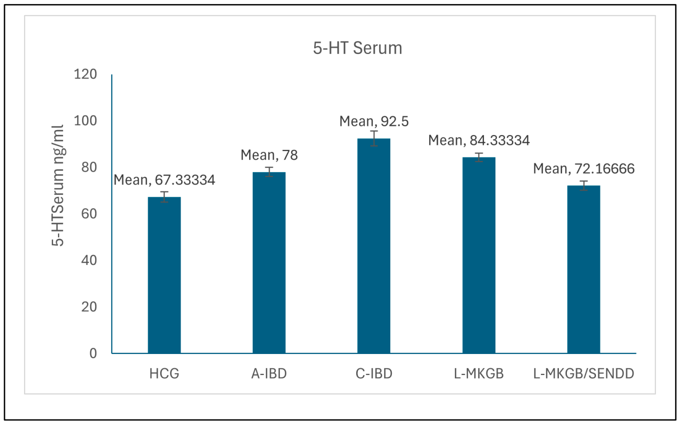

ANOVA

serumHT

	Sum of Squares	df	Mean Square	F	Sig.
Between Groups	2366.467	4	591.617	15.423	.000
Within Groups	959.000	25	38.360		
Total	3325.467	29			

Figure S7. Illustration of ANOVA Statistical Findings Conducted Using SPSS, along with individual mean values for each group regarding 5-HT Levels in Brain and Serum, Respectively

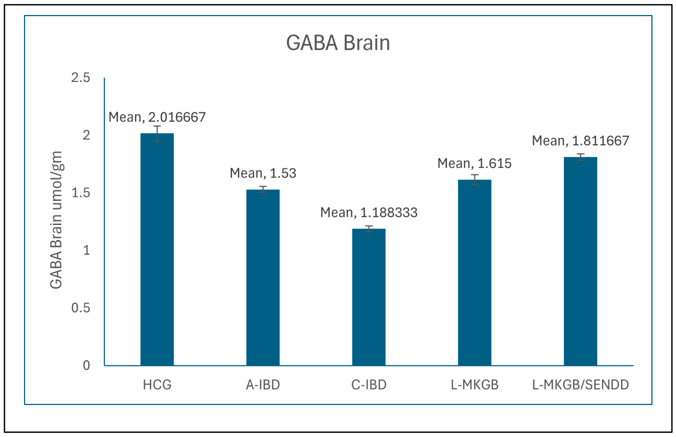

ANOVA

GABAB

	Sum of Squares	df	Mean Square	F	Sig.
Between Groups	2.327	4	.582	50.484	.000
Within Groups	.288	25	.012		
Total	2.615	29			

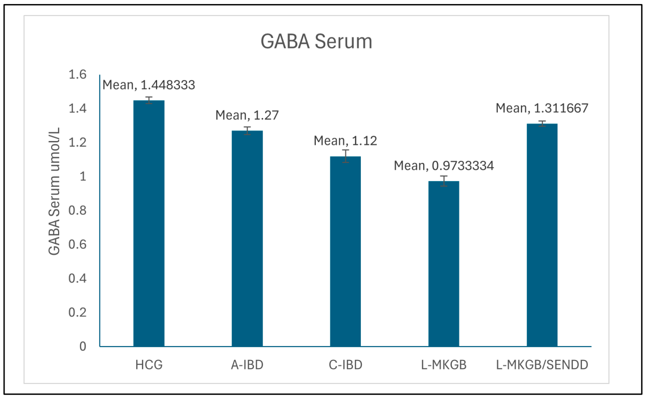

ANOVA

GABAS

	Sum of Squares	df	Mean Square	F	Sig.
Between Groups	.803	4	.201	41.562	.000
Within Groups	.121	25	.005		
Total	.923	29			

Figure S8. Illustration of ANOVA Statistical Findings Conducted Using SPSS, along with individual mean values for each group regarding GABA Levels in Brain and Serum, Respectively.

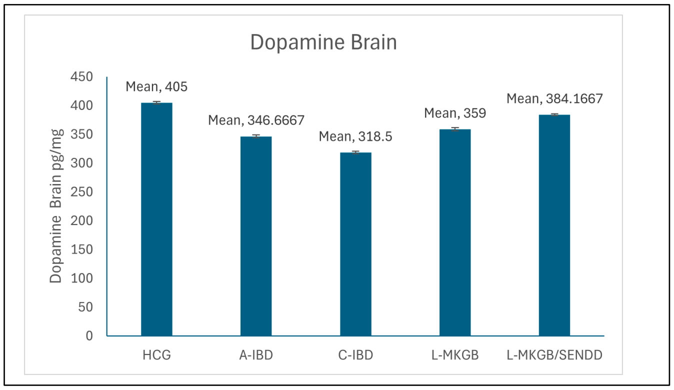

ANOVA

DOPAMINB

	Sum of Squares	df	Mean Square	F	Sig.
Between Groups	26847.000	4	6711.750	195.640	.000
Within Groups	857.667	25	34.307		
Total	27704.667	29			

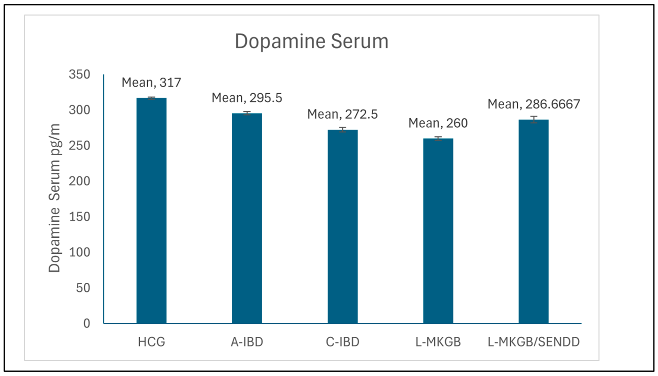

ANOVA

DOPAMINS

	Sum of Squares	df	Mean Square	F	Sig.
Between Groups	11456.333	4	2864.083	50.555	.000
Within Groups	1416.333	25	56.653		
Total	12872.667	29			

Figure S9. Illustration of ANOVA Statistical Findings Conducted Using SPSS, along with individual mean values for each group regarding Dopamine Levels in Brain and Serum, Respectively.

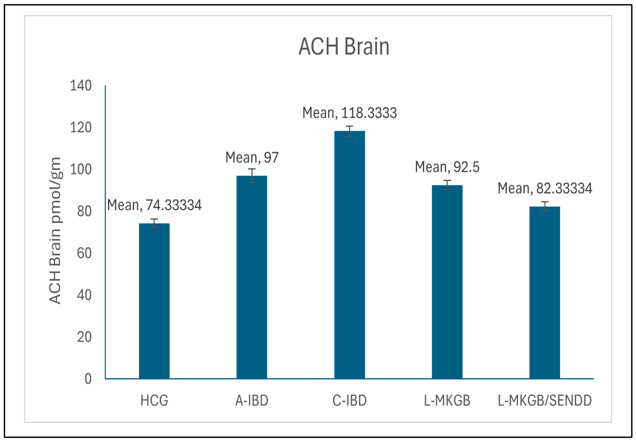

ANOVA

ACHB

	Sum of Squares	df	Mean Square	F	Sig.
Between Groups	6721.200	4	1680.300	45.885	.000
Within Groups	915.500	25	36.620		
Total	7636.700	29			

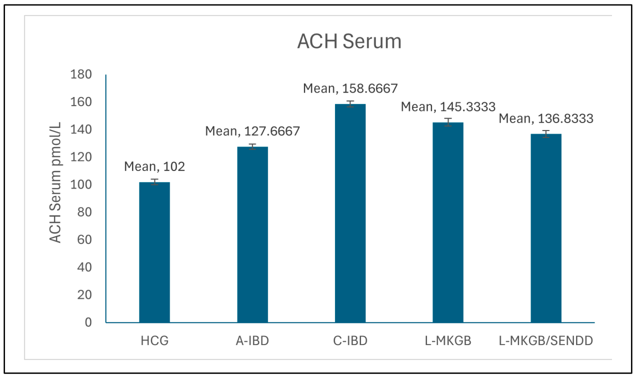

ANOVA

ACHS

	Sum of Squares	df	Mean Square	F	Sig.
Between Groups	10853.867	4	2713.467	84.708	.000
Within Groups	800.833	25	32.033		
Total	11654.700	29			

Figure S10. Illustration of ANOVA Statistical Findings Conducted Using SPSS, along with individual mean values for each group regarding ACH Levels in Brain and Serum, Respectively.

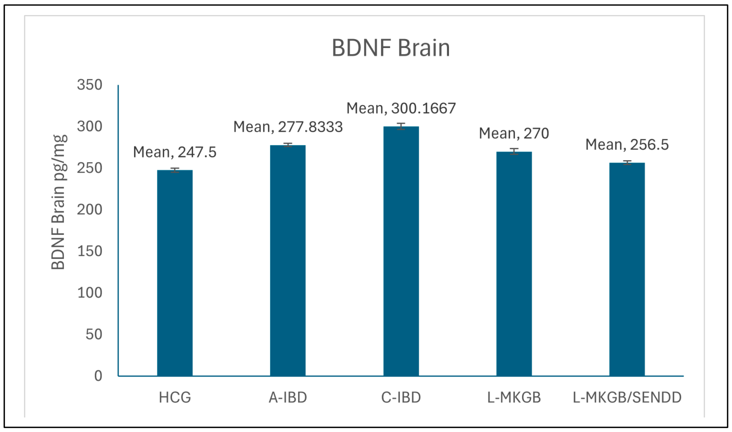

ANOVA

BDNFB

	Sum of Squares	df	Mean Square	F	Sig.
Between Groups	9954.533	4	2488.633	45.590	.000
Within Groups	1364.667	25	54.587		
Total	11319.200	29			

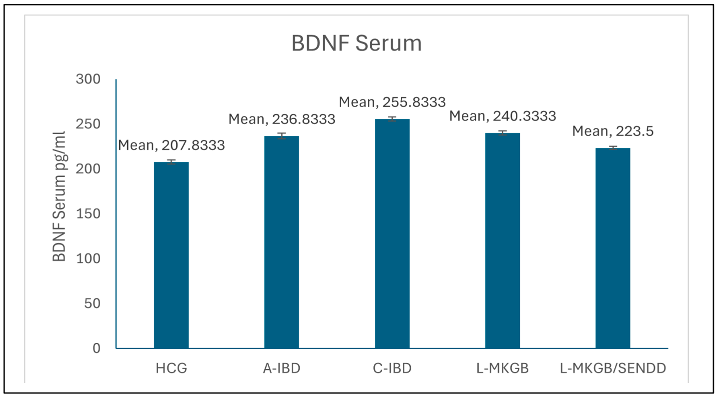

ANOVA

BDNFS

	Sum of Squares	df	Mean Square	F	Sig.
Between Groups	7880.133	4	1970.033	46.846	.000
Within Groups	1051.333	25	42.053		
Total	8931.467	29			

Figure S11. Illustration of ANOVA Statistical Findings Conducted Using SPSS, along with individual mean values for each group regarding BDNF Levels in Brain and Serum, Respectively.

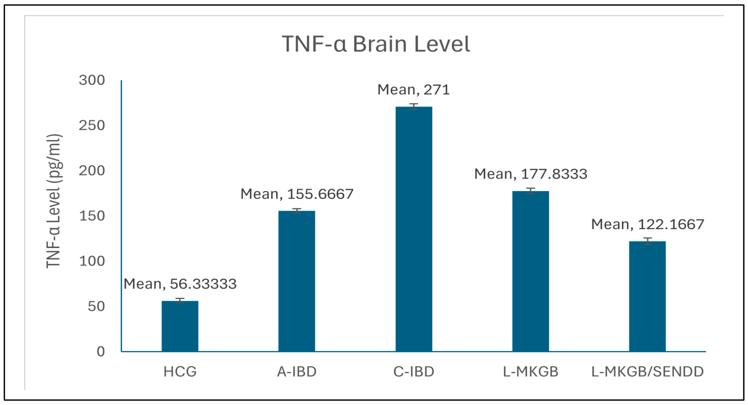

ANOVA

TNFB

	Sum of Squares	df	Mean Square	F	Sig.
Between Groups	148668.867	4	37167.217	725.733	.000
Within Groups	1280.333	25	51.213		
Total	149949.200	29			

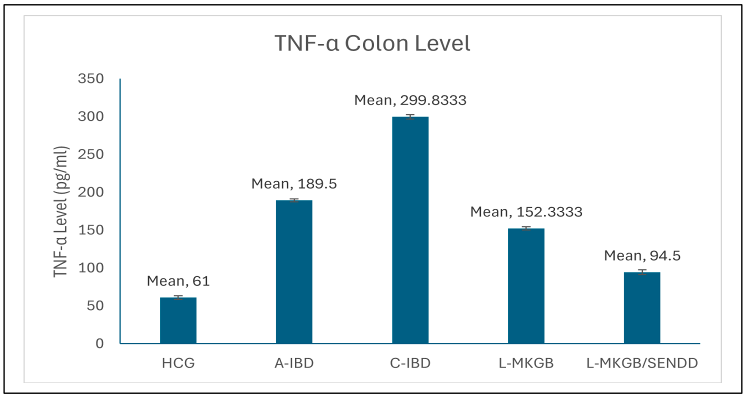

ANOVA

TNFC

	Sum of Squares	df	Mean Square	F	Sig.
Between Groups	207432.200	4	51858.050	1371.664	.000
Within Groups	945.167	25	37.807		
Total	208377.367	29			

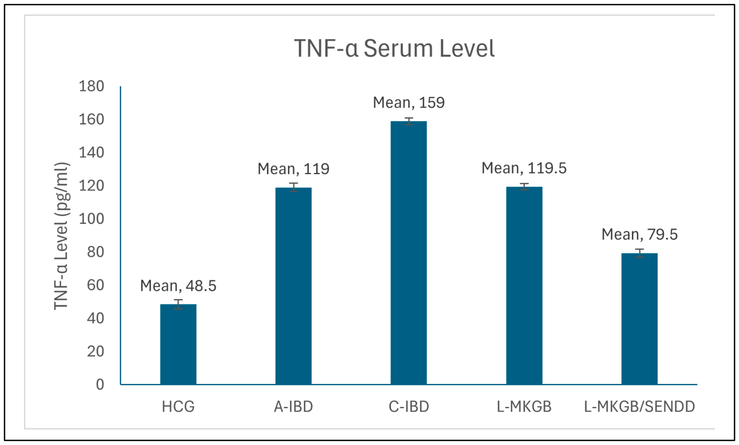

ANOVA

TNFs

	Sum of Squares	df	Mean Square	F	Sig.
Between Groups	42988.200	4	10747.050	332.314	.000
Within Groups	808.500	25	32.340		
Total	43796.700	29			

Figure S12. Illustration of ANOVA Statistical Findings Conducted Using SPSS, along with individual mean values for each group regarding TNF-α Levels in Brain, Colon, and Serum, Respectively.

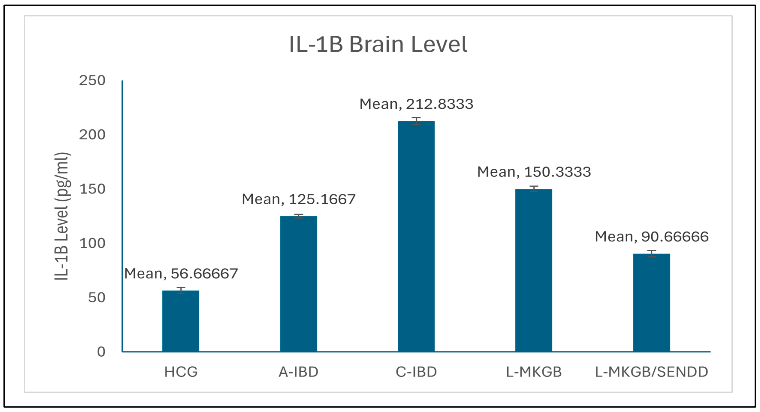

ANOVA

Il1BB

	Sum of Squares	df	Mean Square	F	Sig.
Between Groups	85091.800	4	21272.950	424.892	.000
Within Groups	1251.667	25	50.067		
Total	86343.467	29			

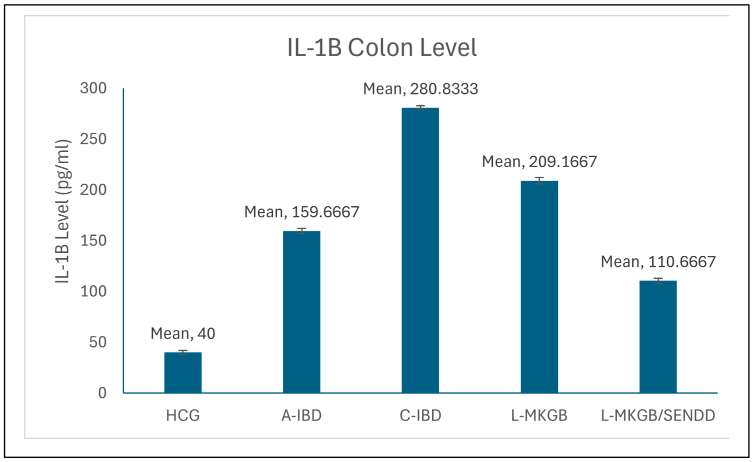

ANOVA

ILBC

	Sum of Squares	df	Mean Square	F	Sig.
Between Groups	203111.533	4	50777.883	1162.143	.000
Within Groups	1092.333	25	43.693		
Total	204203.867	29			

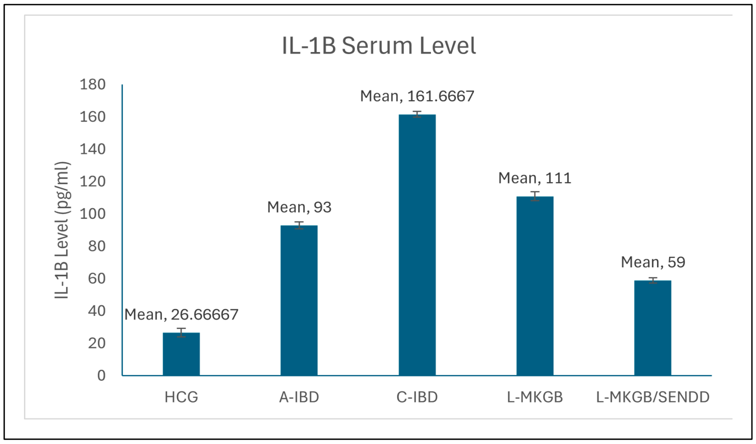

ANOVA

IIBS

	Sum of Squares	df	Mean Square	F	Sig.
Between Groups	63347.200	4	15836.800	438.612	.000
Within Groups	902.667	25	36.107		
Total	64249.867	29			

Figure S13. Illustration of ANOVA Statistical Findings Conducted Using SPSS, along with individual mean values for each group regarding IL-1B Levels in Brain, Colon, and Serum, Respectively.

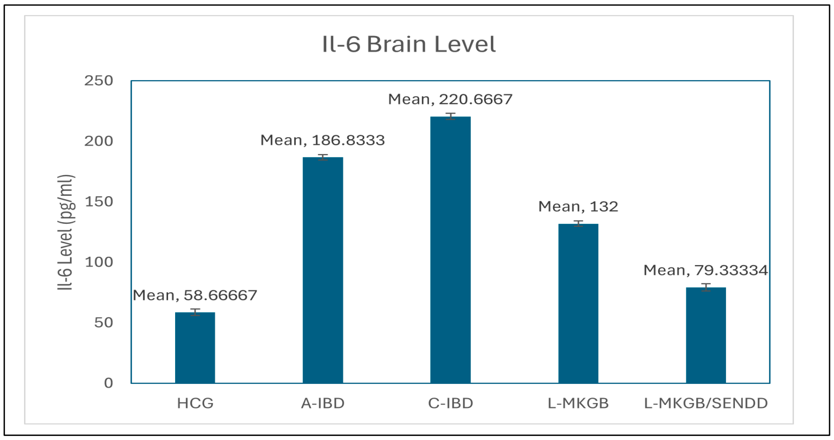

ANOVA

il6B

	Sum of Squares	df	Mean Square	F	Sig.
Between Groups	113752.667	4	28438.167	625.381	.000
Within Groups	1136.833	25	45.473		
Total	114889.500	29			

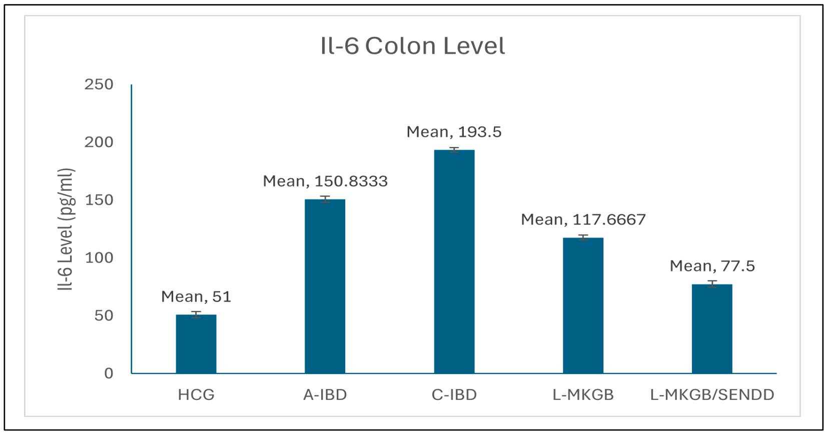

ANOVA

il6C

	Sum of Squares	df	Mean Square	F	Sig.
Between Groups	77445.533	4	19361.383	464.005	.000
Within Groups	1043.167	25	41.727		
Total	78488.700	29			

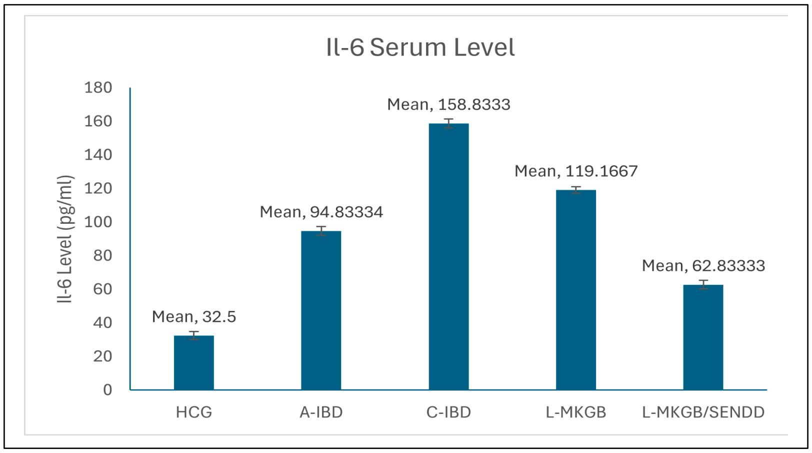

ANOVA

il6S

	Sum of Squares	df	Mean Square	F	Sig.
Between Groups	57542.133	4	14385.533	330.297	.000
Within Groups	1088.833	25	43.553		
Total	58630.967	29			

Figure S14. Illustration of ANOVA Statistical Findings Conducted Using SPSS, along with individual mean values for each group regarding IL-6 Levels in Brain, Colon, and Serum, Respectively.

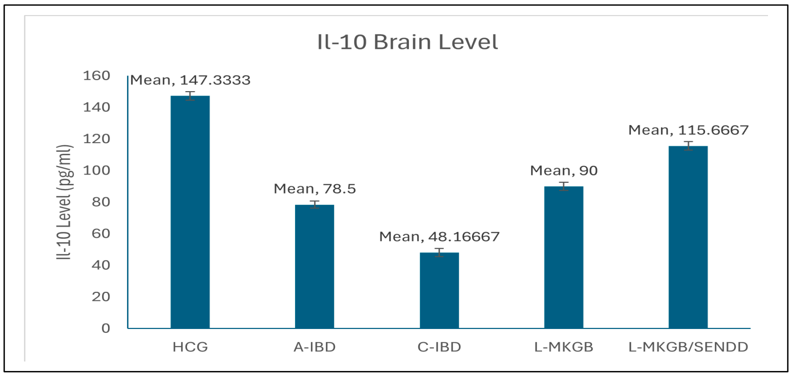

ANOVA

Il10B

	Sum of Squares	df	Mean Square	F	Sig.
Between Groups	33912.867	4	8478.217	180.081	.000
Within Groups	1177.000	25	47.080		
Total	35089.867	29			

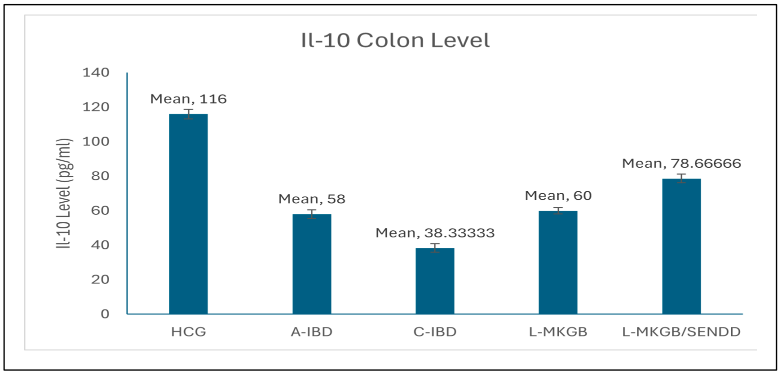

ANOVA

il10C

	Sum of Squares	df	Mean Square	F	Sig.
Between Groups	20626.133	4	5156.533	121.311	.000
Within Groups	1062.667	25	42.507		
Total	21688.800	29			

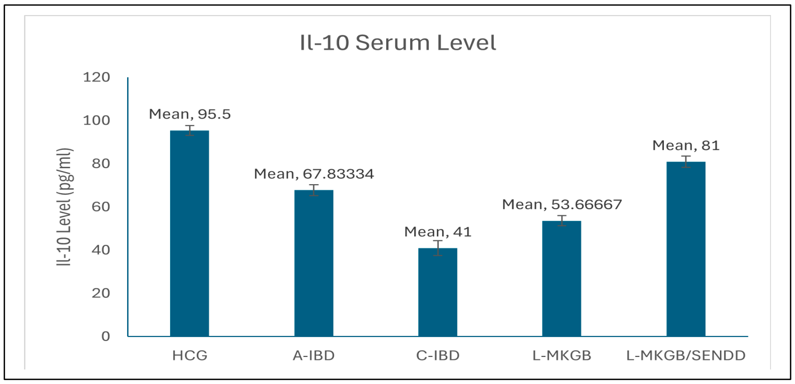

ANOVA

il10S

	Sum of Squares	df	Mean Square	F	Sig.
Between Groups	11157.133	4	2789.283	54.238	.000
Within Groups	1285.667	25	51.427		
Total	12442.800	29			

Figure S15. Illustration of ANOVA Statistical Findings Conducted Using SPSS, along with individual mean values for each group regarding IL-10 Levels in Brain, Colon, and Serum, Respectively.

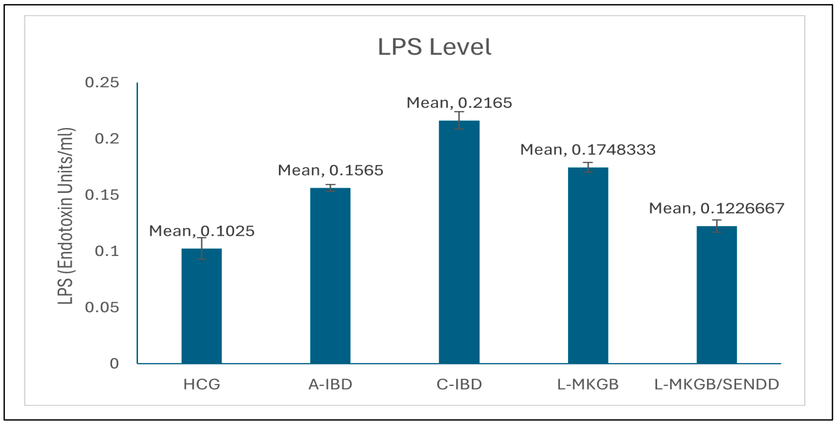

ANOVA

LPS

	Sum of Squares	df	Mean Square	F	Sig.
Between Groups	.048	4	.012	47.322	.000
Within Groups	.006	25	.000		
Total	.054	29			

Figure S16. Illustration of ANOVA statistical findings conducted using SPSS, along with individual mean values for each group regarding LPS.

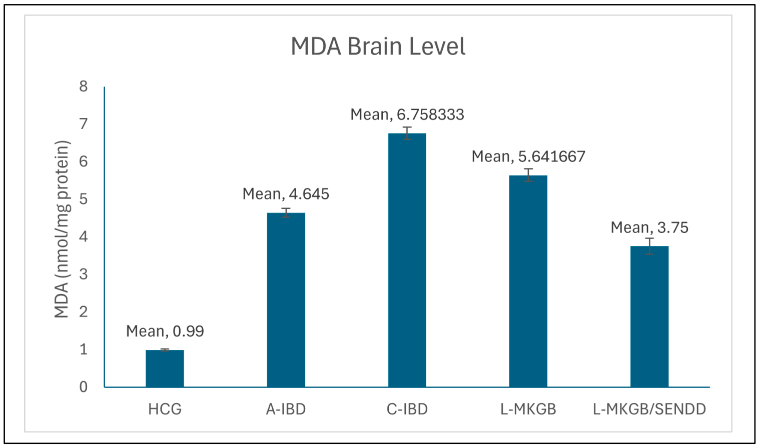

ANOVA

MDAB

	Sum of Squares	df	Mean Square	F	Sig.
Between Groups	115.229	4	28.807	210.314	.000
Within Groups	3.424	25	.137		
Total	118.653	29			

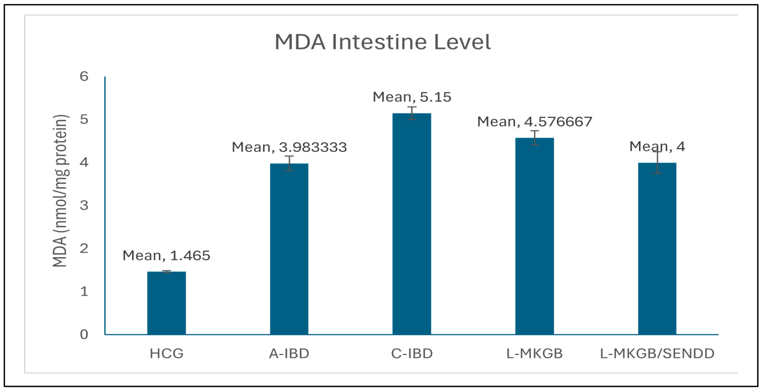

ANOVA

MDAI

	Sum of Squares	df	Mean Square	F	Sig.
Between Groups	47.673	4	11.918	71.441	.000
Within Groups	4.171	25	.167		
Total	51.843	29			

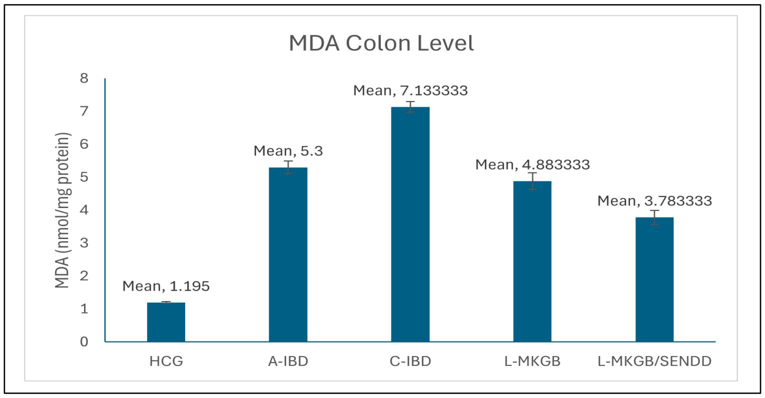

ANOVA

MDAC

	Sum of Squares	df	Mean Square	F	Sig.
Between Groups	114.898	4	28.724	136.602	.000
Within Groups	5.257	25	.210		
Total	120.155	29			

Figure S17. Illustration of ANOVA Statistical Findings Conducted Using SPSS, along with individual mean values for each group regarding MDA Levels in Brain, Intestine, and Colon, Respectively.

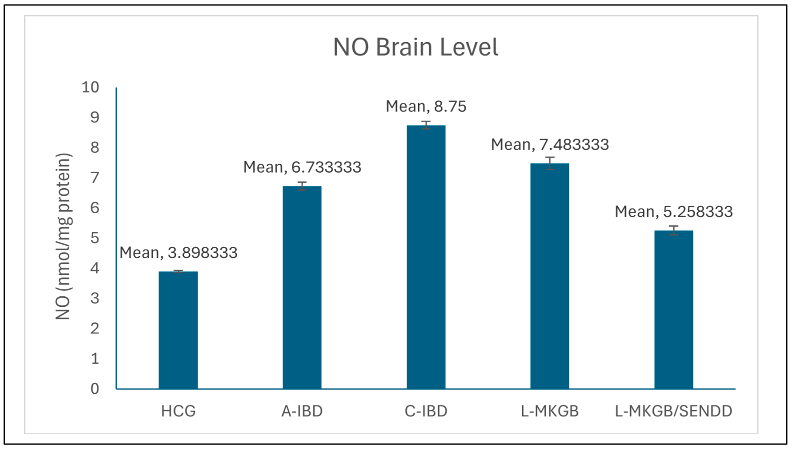

ANOVA

NOB

	Sum of Squares	df	Mean Square	F	Sig.
Between Groups	86.196	4	21.549	153.489	.000
Within Groups	3.510	25	.140		
Total	89.705	29			

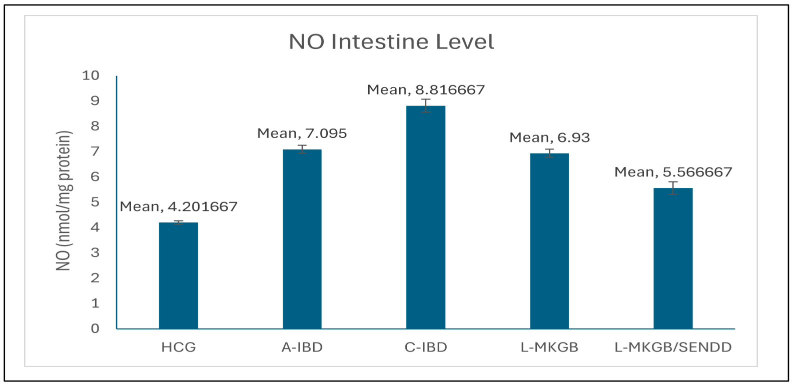

ANOVA

NOI

	Sum of Squares	df	Mean Square	F	Sig.
Between Groups	67.776	4	16.944	83.177	.000
Within Groups	5.093	25	.204		
Total	72.869	29			

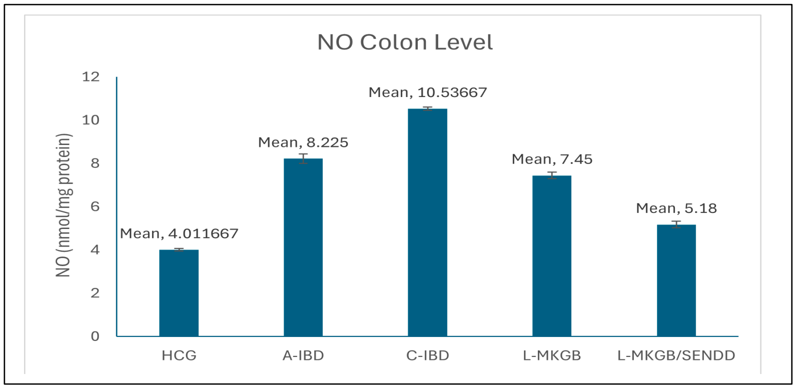

ANOVA

NOC

	Sum of Squares	df	Mean Square	F	Sig.
Between Groups	167.205	4	41.801	206.101	.000
Within Groups	5.070	25	.203		
Total	172.276	29			

Figure S18. Illustration of ANOVA Statistical Findings Conducted Using SPSS, along with individual mean values for each group regarding NO Levels in Brain, Intestine, and Colon, Respectively.

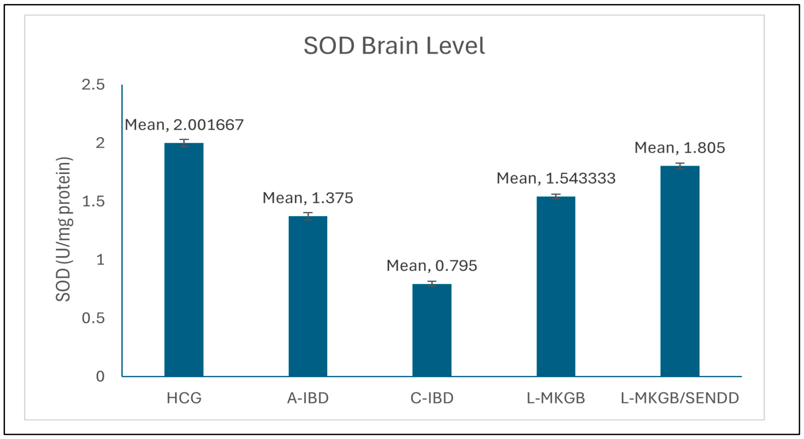

ANOVA

SODB

	Sum of Squares	df	Mean Square	F	Sig.
Between Groups	5.155	4	1.289	315.037	.000
Within Groups	.102	25	.004		
Total	5.257	29			

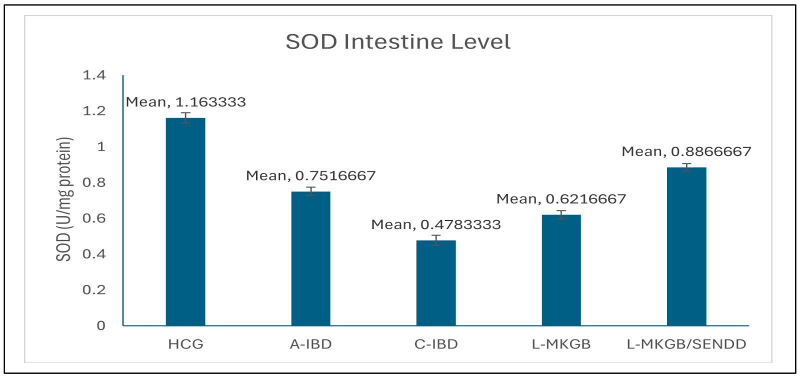

ANOVA

SODI

	Sum of Squares	df	Mean Square	F	Sig.
Between Groups	1.651	4	.413	108.726	.000
Within Groups	.095	25	.004		
Total	1.746	29			

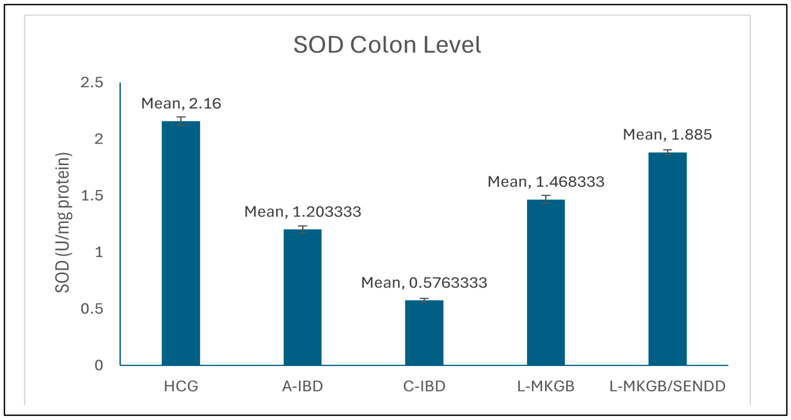

ANOVA

SODC

	Sum of Squares	df	Mean Square	F	Sig.
Between Groups	9.105	4	2.276	448.448	.000
Within Groups	.127	25	.005		
Total	9.231	29			

Figure S19. Illustration of ANOVA Statistical Findings Conducted Using SPSS, along with individual mean values for each group regarding SOD Levels in Brain, Intestine, and Colon, Respectively.

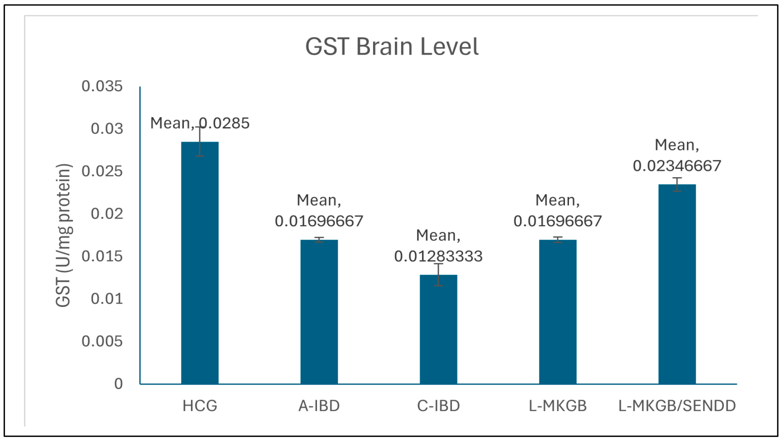

ANOVA

GSTB

	Sum of Squares	df	Mean Square	F	Sig.
Between Groups	.001	4	.000	35.243	.000
Within Groups	.000	25	.000		
Total	.001	29			

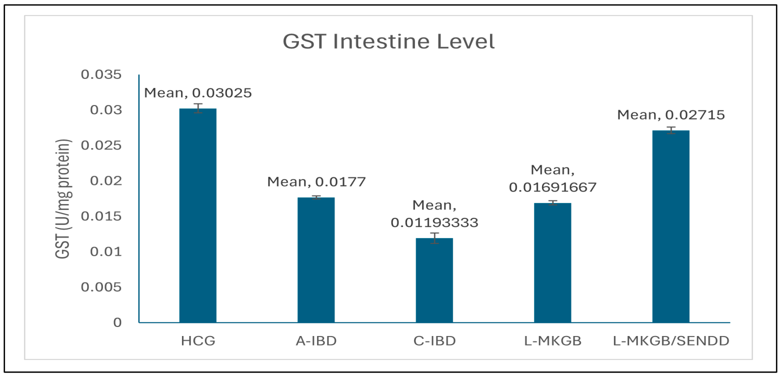

ANOVA

GSTI

	Sum of Squares	df	Mean Square	F	Sig.
Between Groups	.001	4	.000	231.994	.000
Within Groups	.000	25	.000		
Total	.001	29			

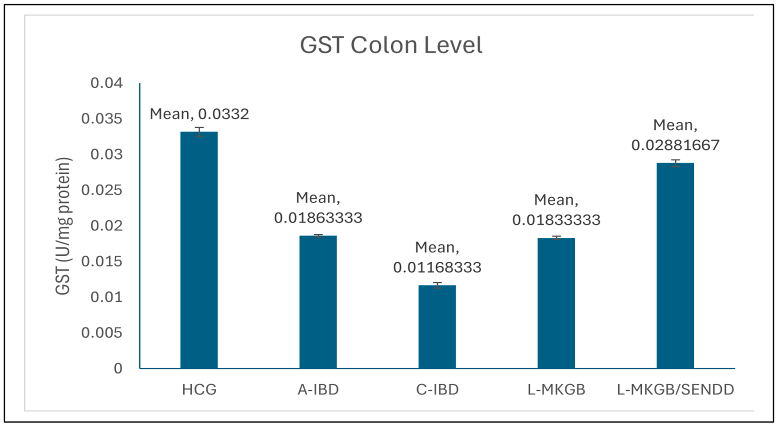

ANOVA

GSTC

	Sum of Squares	df	Mean Square	F	Sig.
Between Groups	.002	4	.000	443.835	.000
Within Groups	.000	25	.000		
Total	.002	29			

Figure S20. Illustration of ANOVA Statistical Findings Conducted Using SPSS, along with individual mean values for each group regarding GST Levels in Brain, Intestine, and Colon, Respectively.

4) Particle size distribution and zeta potential analysis

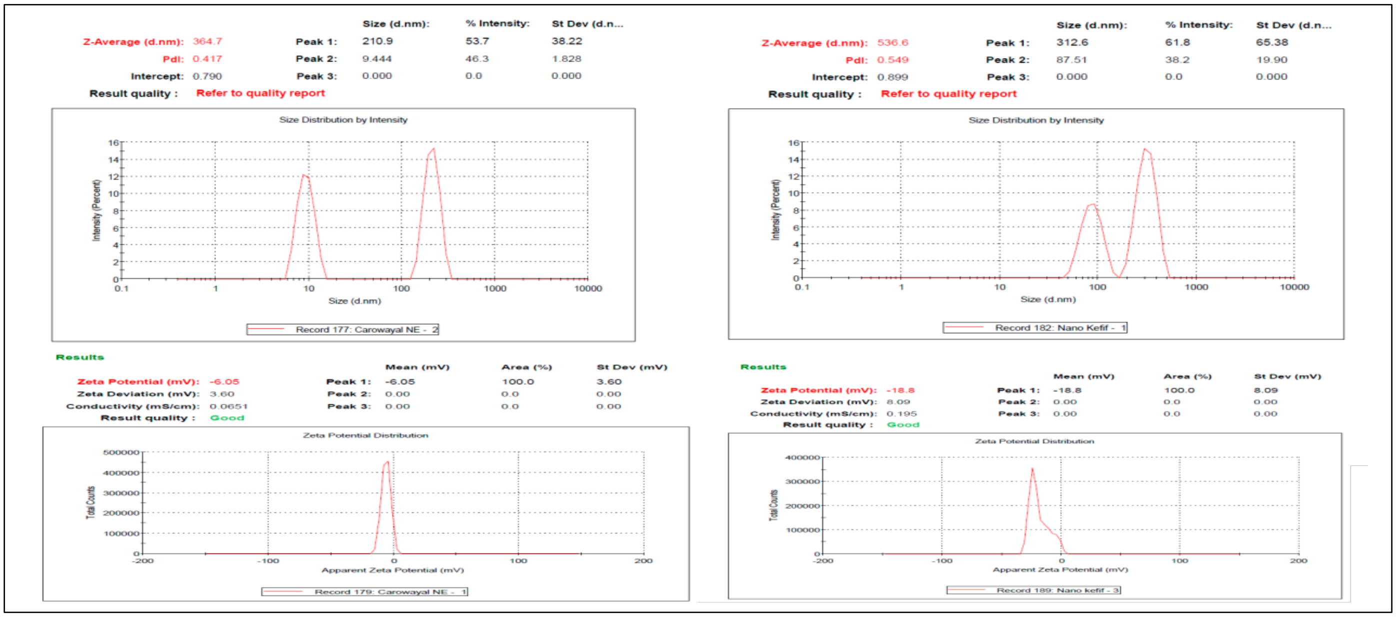

Figure S21. Particle size distribution and zeta potential analysis of licorice-SNESNS and lyophilized Kefir-loaded licorice-SNESNS formulations. The particle size analysis illustrates the size distribution of the nanoemulsion and nanosuspension particles, indicating the formulation's homogeneity and stability. Zeta potential measurements provide insight into the surface charge and colloidal stability of the formulations, with higher absolute values suggesting greater stability against aggregation.
